# Antifungal Prenylated Diphenyl Ethers from *Arthrinium arundinis*, an Endophytic Fungus Isolated from the Leaves of Tobacco (*Nicotiana tabacum* L.)

**DOI:** 10.3390/molecules23123179

**Published:** 2018-12-02

**Authors:** Peng Zhang, Xin Li, Xiao-Long Yuan, Yong-Mei Du, Bin-Gui Wang, Zhong-Feng Zhang

**Affiliations:** 1 Tobacco Research Institute, Chinese Academy of Agricultural Sciences, Qingdao 266101, China; zhangpeng@caas.cn (P.Z.); rayrock@126.com (X.-L.Y.); duyongmei@caas.cn (Y.-M.D.); 2 Laboratory of Marine Biology and Biotechnology, Qingdao National Laboratory for Marine Science and Technology, Key Laboratory of Experimental Marine Biology, Institute of Oceanology, Chinese Academy of Sciences, Qingdao 266071, China; lixin871014@163.com

**Keywords:** tobacco-derived endophytic fungus, *Arthrinium arundinis*, *Nicotiana tabacum* L., prenylated diphenyl ethers, antifungal activity, cytotoxicity

## Abstract

An endophytic fungus *Arthrinium arundinis* TE-3 was isolated and purified from the fresh leaves of cultivated tobacco (*Nicotiana tabacum* L.). Chemical investigation on this fungal strain afforded three new prenylated diphenyl ethers (**1**–**3**) as well as three known analogues (**4**–**6**). Structure elucidation of the isolated compounds was carried out by analysis of 1D and 2D nuclear magnetic resonance (NMR) and high-resolution electrospray ionization mass spectroscopy (HRESIMS) spectra, as well as by comparison of those data with literature data. The absolute configuration of the stereogenic center at C-8 in **1** was assigned by comparison of the experimental and calculated ECD spectra. Compounds **1** and **2** showed selective antifungal activity against *Mucor hiemalis* with minimum inhibitory concentration (MIC) values of 8 and 4 μg/mL, respectively. Compounds **5** and **6** exhibited inhibitory activity against *Alteraria alternata* with an MIC value of 8 μg/mL. In the cytotoxic assay, **2**, **5**, and **6** displayed moderate in vitro cytotoxicity against the human monocytic cell line (THP-1 cell line), with IC_50_ values of 40.2, 28.3, and 25.9 μM, respectively. This study indicated that endophytic fungi possess great potential for exploring new bioactive secondary metabolites.

## 1. Introduction

**Figure 1:**
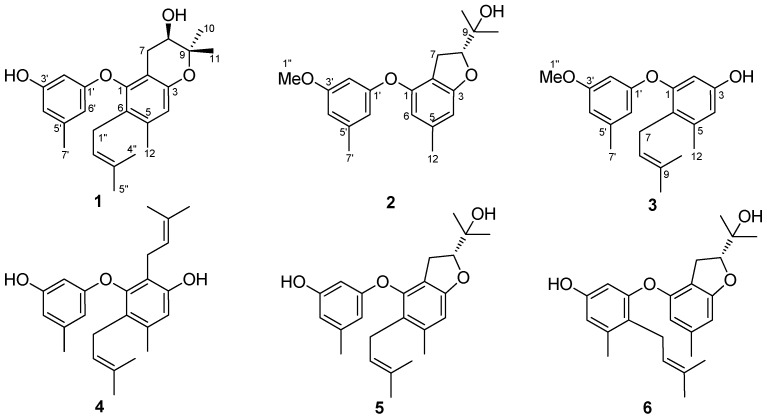
Structures of compounds **1**–**6.**

Endophytic microorganisms are those that inhabit at the internal living tissues of plants without causing visible damage to their hosts [1]. Endophytes are commonly found in almost every plant, and simultaneously each individual plant is host to one or more endophytes [1,2]. In recent decades, endophytes have attracted great attention due to their potential to produce extensive bioactive secondary metabolites with prominent medicinal and/or agricultural applications [3,4,5]. During our continuous research to discover structurally novel secondary metabolites from endophytic fungi, especially in the searching for those with promising cytotoxic and antifungal activities [6,7,8], we isolated and purified an endophytic fungus *Arthrinium arundinis* TE-3 from the fresh leaves of cultivated tobacco (*Nicotiana tabacum* L.). Chemical and biological investigations on this fungal strain have led to the isolation and identification of three new prenylated diphenyl ethers (**1**–**3**) and previously described compounds (**4**–**6**) (Figure 1). These compounds incorporate one or more prenyl groups, and some of which were modified as isopentenyl, dihydrofuran, and dihydropyran moieties [9,10]. Herein, we report the isolation and structure elucidation of these diverse prenylated diphenyl ethers, and evaluated the antifungal and cytotoxic activities of the isolated compounds.

## 2. Results and Discussion

### 2.1. Structural Elucidation of the New Compounds

**Figure 2:**
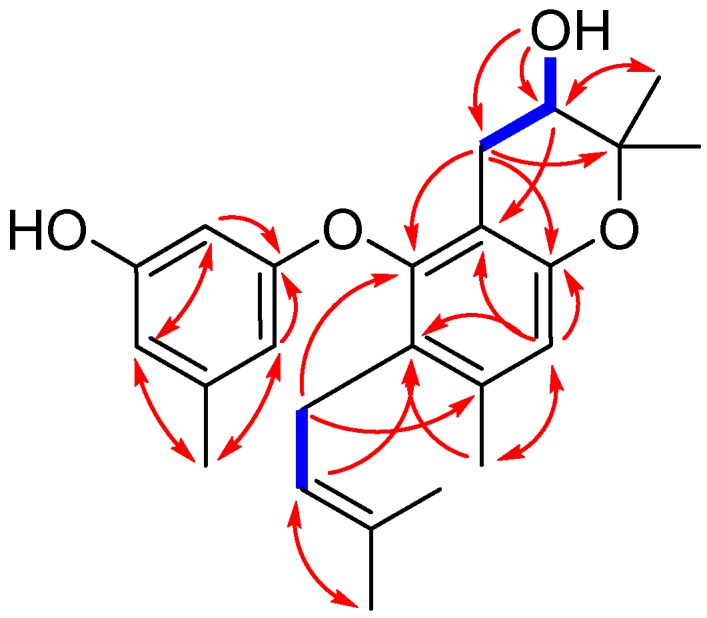
Key COSY (bold blue lines) and HMBC (red arrows) correlations for compound **1**.

Compound **1** was isolated as yellowish oil. Its molecular formula was determined to be C_24_H_30_O_4_, as evidenced from the quasimolecular ion peak at m/z 381.2076 [M − H]^−^ (calcd. for C_24_H_29_O_4_, 381.2071) in its HRESIMS (Figure S1 in the Supplementary Material). Its ^1^H-NMR data (Table 1 and Figure S2) as well as the HSQC (Heteronuclear Singular Quantum Correlation) data (Figure S5) displayed signals that were assigned to one prenyl group at δ_H_ 3.05 (2H, br d, *J* = 6.8 Hz, H-1″), 4.92 (1H, t, *J* = 6.8 Hz, H-2″), and 1.56 (6H, br s, H-4″ and H-5″), one prenyl-derived group at δ_H_ 2.55 (1H, dd, *J* = 17.0, 5.4 Hz, H-7a), 2.19 (1H, m, H-7b), 3.52 (1H, dt, *J* = 8.0, 5.4 Hz, H-8), 1.23 (3H, s, H-10), and 1.08 (3H, s, H-11), two additional singlet methyls at δ_H_ 2.18 (3H, s, H-12) and 2.14 (3H, s, H-7′), four isolated aromatic methines at δ_H_ 6.51 (1H, s, H-4), 5.91 (1H, s, H-2′), 6.20 (1H, s, H-4′), and 6.06 (1H, s, H-6′), and two exchangeable protons at δ_H_ 9.30 (1H, s, 3′-OH) and 5.07 (1H, s, d, *J* = 4.8 Hz, 8-OH). The ^13^C-NMR and DEPT (Distortionless Enhancement by Polarization Transfer) spectra (Table 1 and Figure S3) revealed the presence of 24 carbons, which were classified into six methyls, two methylenes, six methines (including five aromatic/olefinic and one oxygenated sp^3^ carbons), and ten non-protonated carbons (including one oxygenated sp^3^ carbon). These above data demonstrated that **1** contains a diphenyl ether unit belonging to the diorcinol family [10], which was concluded by the HMBC (Heteronuclear Multiple Bond Correlation) correlations from H-2′ to C-1′, C-4′, and C-6′, from H-6′ to C-1′ and C-4′, and C-7′, and from H-4 to C-2, C-3, C-6, and C-12 (Figure 2). The 1D and 2D NMR data of compound **1** were similar to those of compound **5**, which was previously isolated from the mantis-associated fungus *Aspergillus versicolor* [9]. The main differences were the two oxygen-bearing carbons resonating at C-8 and C-9. The chemical shifts of C-8 and C-9 in **1** were 68.3 and 77.1 ppm, whereas in **5**, they were 89.6 and 70.2 ppm, respectively. The upfield shift of C-8 in **1** indicated that the dihydrofuran ring may transform into dihydropyran ring system, which was reinforced by the observation of a doublet exchangeable proton (8-OH), the COSY correlations from H-8 to 8-OH and H-7, as well as the key HMBC correlations from 8-OH to C-7, C-8, and C-9 and from H-8 to C-2 (Figure 2 and Figure S6). This deduction and variation was also verified through analysis of its acetylated product by Lin et al. [11]. Thus, the planar structure of **1** was determined and a trivial name, diorcinol M, was assigned.

**Figure 3:**
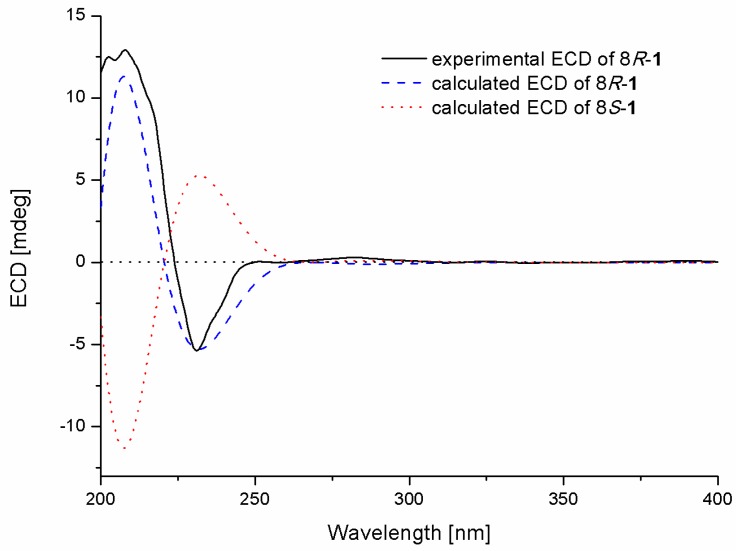
Experimental and calculated ECD spectra of compound **1**.

The absolute configuration of the stereogenic center at C-8 in **1** was determined by comparison of the experimental and calculated ECD spectra in Gaussian 09 [12]. The minimum energy conformers were obtained by geometry optimization of each possible isomer of **1**, and the TDDFT (Time-Dependent Density Functional Theory) method was then employed at PBE0/TZVP level to get calculated ECD spectra of **1**. The experimental ECD spectrum of **1** displayed excellent accordance with that of calculated for 8*R*-**1** at this level, which allowed unambiguous assignment of its absolute configuration (Figure 3).

Compound **2** was obtained as yellowish oil, and its molecular formula was determined to be C_20_H_24_O_4_ by HRESIMS data (Figure S7). The ^1^H and ^13^C-NMR data (Figures S8 and S9) of **2** revealed that the structural feature was very similar to that of a dihydrobenzofuran derivative [9,13], except for an additional methoxy group (δ_H_/δ_C_ 3.68/55.6, H/C-1″) observed in **2**. The methoxy group was located at C-3′ by the HMBC correlation from H-1″ to C-3′ (Figure S12). Compound **2** was named as diorcinol N. Compound **2** also had only one chiral center at C-8, thus **2** was proposed to have the same 8*R* configuration as the known compound (the compound name was not given) by the similar negative specific rotation data (${\left[\alpha \right]}_{D}^{25}$ −17.4 (c 0.05, MeOH) for **2**) [9]. Moreover, the similarity of the ECD spectra of **2** and the previously described compound verified this assignment (Figure S19).

The molecular formula of compound **3** was established as C_20_H_24_O_3_ using HRESIMS data (Figure S13). Examining its ^1^H and ^13^C-NMR spectra (Figures S14 and S15) showed close similarity to those of diorcinol D [10], except for a methoxy group substitued at C-3′. The key HMBC correlation from H-1″ (δ_H_ 3.67) to C-3′ (δ_C_ 160.8) supported this deduction and confirmed its location (Figure S18). Thus, compound **3** was elucidated as the C-3′ *O*-methyl derivative of diorcinol D, and a trivial name of diorcinol O was assigned to this compound.

### 2.2. Biological Activities of the Isolated Compounds

Prenylated diphenyl ethers have been previously reported to possess antimicrobial, cytotoxic, antioxidant, and antiviral activities [8,9,10]. Therefore, the antifungal activity (Table 2) against six commonly occurring plant-pathogenic fungi (*Alternaria alternata*, *Cochliobolus heterostrophus*, *Gaeumannomyces graminis*, *Glomerella cingulata*, *Mucor hiemalis*, and *Thielaviopsis basicola*) and cytotoxicity against four tumor cell lines (A549, HeLa, MCF-7, and THP-1) of compounds **1**–**6** were evaluated. Compounds **1** and **2** showed selective antifungal activity against *M. hiemalis* with MIC values of 8 and 4 μg/mL, respectively. Compounds **5** and **6** exhibited inhibitory activity against *A. alternata* with an MIC value of 8 μg/mL. It should be pointed out that *A. alternata* is an important fungal disease in tobacco, and it can cause tobacco red spot disease. The isolation of these antifungal secondary metabolites implied that tobacco-derived endophytic fungi may be a new resource with huge development prospect and latent capacity. Moreover, in the cytotoxic assay, compounds **2**, **5**, and **6** displayed weak to moderate in vitro cytotoxicity against the THP-1 cell line, with IC_50_ values of 40.2, 28.3, and 25.9 μM, respectively, whereas others showed weak or no activities against other cell lines (IC_50_ > 50 μM, data were not shown).

## 3. Materials and Methods

### 3.1. General Experimental Procedures

Optical rotations were determined using a Jasco P-1020 digital polarimeter (Jasco, Tokyo, Japan). UV spectra were recorded using a Shimadzu UV-2700 spectrophotometer (Shimadzu, Kyoto, Japan). ECD spectra were obtained with a Jasco J-815-150S circular dichroism spectrometer (Jasco, Inc., Tokyo, Japan). The NMR spectra were recorded using an Agilent DD2 500 MHz NMR spectrometer (500 MHz for ^1^H and 125 MHz for ^13^C, Agilent, Santa Clara, CA, USA). HRESIMS data were obtained using an LTQ Orbitrap XL spectrometer (Thermo Scientific, Waltham, MA, USA). Column chromatography was performed using silica gel (200–300 mesh, Qingdao Haiyang Chemical Factory, Qingdao, China), Lobar LiChroprep RP-18 (40–60 μm, Merck, Darmstadt, Germany), and Sephadex LH-20 (Merck, Darmstadt, Germany).

### 3.2. Fungal Isolation and Identification

The fungal strain AT-3 was firstly isolated from the fresh leaves of cultivated tobacco (*Nicotiana tabacum* L.), which was collected from the Modern Tobacco Agricultural Science and Technology Demonstration Garden on Wangcheng Slope, Enshi, Hubei, in August 2016. The fresh leaves were kept in a plastic case at 4 °C and were handled within 24 h. The surfaces of the leaves were sterilized with 75% ethanol for 1 min, 2.5% sodium hypochlorite for 30 s, and subsequent 75% ethanol for 1 min. Then the aseptic leaves were washed with sterilized distilled water and were cut into small pieces (approximately 0.5 × 0.5 cm). Some of pieces were put into PDA medium (Solarbio, Beijing, China). Fungal strains were grown out from small leaf tissues after three to five days’ incubation. The fungal strain AT-3 was picked up and purified with repeated streak cultivation. The fungus AT-3 was identified as *Arthrinium arundinis* with GenBank number of MK182939 based on the sequence of the ITS region. The voucher strain was deposited in the China General Microbiological Culture Collection Center with the CGMCC number 14792.

### 3.3. Fermentation, Extraction and Isolation

The fungus was statically cultured in liquid PDB medium (Potato Dextrose Broth, Solarbio, Beijing, China) in 1000 mL Erlenmeyer flasks (each flask containing 300 mL medium) at 28 °C for 30 days. The broth (a total of 200 flasks) was extracted exhaustively with equivoluminal EtOAc. After evaporated under reduced pressure, the extracts were condensed to yield 26 g of residue. The residue was then subjected to silica gel column chromatography with mixed petroleum ether (PE)-EtOAc (from 10:1 to 1:1) and dichloromethane (DCM)-MeOH (from 20:1 to 1:1) to give eight fractions (Fr. A-Fr. H). Fr. D (2.0 g), eluted with PE-EtOAc 1:1, was applied to column chromatography on Lobar LiChroprep RP-18 with a MeOH-H_2_O gradient (from 1:9 to 1:0) to give five subfractions (Fr. D1-Fr. D5). Fr. D2 (120 mg) was separated by silica gel (DCM-MeOH 20:1, *v*/*v*) to yield compound **2** (16.8 mg) and **6** (3.5 mg). Fr. D3 (80 mg) was subjected to Sephadex LH-20 (MeOH) and then silica gel (DCM-MeOH 20:1, *v*/*v*) to yield compound **3** (10.2 mg). Fr. E (2.6 g), eluted with DCM-MeOH 20:1, was applied to Lobar LiChroprep RP-18 with a MeOH-H_2_O gradient (from 1:9 to 1:0) to give six subfractions (Fr. E1-Fr. E6). Fr. E2 (131 mg) was chromatographed on Sephadex LH-20 (MeOH) to yield compound **1** (27.0 mg). Fr. E4 (64 mg) was purified on silica gel (DCM-acetone 10:1, *v*/*v*) to yield compound **5** (4.4 mg). Fr. E5 (200 mg) was separated using silica gel (DCM-MeOH 30:1, *v*/*v*) and then Sephadex LH-20 (MeOH) to yield compound **4** (32.0 mg).

Diorcinol M (**1**): yellowish oil; ${\left[\alpha \right]}_{D}^{25}$ −12.8 (*c* 0.04, MeOH); UV (MeOH) *λ*_max_ (log *ε*) 205 (4.64), 282 (3.29) nm; ECD (MeOH) 208 (+12.92), 231 (−5.38); ^1^H and ^13^C-NMR data (in DMSO-*d*_6_, 500 and 125 MHz), see Table 1; HRESIMS *m*/*z* 381.2076 [M − H]^−^ (calcd for C_24_H_29_O_4_, 381.2071).

Diorcinol N (**2**): yellowish oil; ${\left[\alpha \right]}_{D}^{25}$ −17.4 (*c* 0.05, MeOH); UV (MeOH) *λ*_max_ (log *ε*) 207 (4.82), 280 (3.62) nm; ^1^H and ^13^C NMR data (in DMSO-*d*_6_, 500 and 125 MHz), see Table 1; HRESIMS *m*/*z* 327.1597 [M − H]^−^ (calcd for C_20_H_23_O_4_, 327.1602).

Diorcinol O (**3**): yellowish oil; UV (MeOH) *λ*_max_ (log *ε*) 205 (4.97), 281 (3.74) nm; ^1^H and ^13^C NMR data (in DMSO-*d*_6_, 500 and 125 MHz), see Table 1; HRESIMS *m*/*z* 311.1651 [M − H]^−^ (calcd for C_20_H_23_O_3_, 311.1653).

### 3.4. Antifungal Assay

The antifungal assay of the isolated compounds was performed using previously reported method [14]. Six commonly occurring plant-pathogenic fungi (*Alternaria alternata*, *Cochliobolus heterostrophus*, *Gaeumannomyces graminis*, *Glomerella cingulata*, *Mucor hiemalis*, and *Thielaviopsis basicola*) were selected for the assay. The results were indicated by MIC values, which were defined as the minimal detectable concentration with no obvious growth after the incubation. Prochloraz, a broad-spectrum fungicide commonly used in agriculture, was used as a positive control.

### 3.5. Cytotoxicity Assay

The cytotoxicity of compounds **1**–**6** was assessed using the Cell Counting Kit (CCK)-8 (Dojindo, Kumamoto, Japan) method [15] with the following human tumor cell lines: A549 (human lung cancer cell line), HeLa (human cervical cancer cell line), MCF-7 (human breast cancer cell line), and THP-1 (human monocytic cell line).

### 3.6. Computational Section

Conformational searches were performed via molecular mechanics using the MM+ method in HyperChem software (Version 8.0, Hypercube, Inc., Gainesville, FL, USA), and the geometries were further optimized at the B3LYP/6-31G(d) PCM/MeOH level via Gaussian 09 software (Version D.01; Gaussian, Inc.:Wallingford, CT, USA) [12] to give the energy-minimized conformers. Then, the optimized conformers were subjected to the calculations of ECD spectra using TDDFT at PBE0/TZVP. Solvent effects of the MeOH solution were evaluated at the same DFT level using the SCRF/PCM method.

## 4. Conclusions

In summary, three new prenylated diphenyl ethers namely diorcinol M-O (**1**–**3**) were isolated from the endophytic fungus *Arthrinium arundinis* TE-3 purified from the leaves of cultivated tobacco. Their antifungal activity as well as the cytotoxicity was assessed. Compounds **1** and **2** showed inhibitory activity against *Mucor hiemalis* with MIC values of 8 and 4 μg/mL, while most importantly, compounds **5** and **6** exhibited promising activity against *Alteraria alternata* with an MIC value of 8 μg/mL. Some of the isolated compounds showed moderate cytotoxicity. This study indicated that the tobacco-derived endophytic fungi possess great potential for exploring new bioactive secondary metabolites.

## Figures and Tables

**Table 1:** ^1^H (500 MHz) and ^13^C-NMR (125 MHz) data of compounds **1**–**3** in DMSO-*d*_6_.

No.	Compound 1		Compound 2		Compound 3	
	*δ*_H_ (mult, *J* in Hz)	*δ*_C_, type	*δ*_H_ (mult, *J* in Hz)	*δ*_C_, type	*δ*_H_ (mult, *J* in Hz)	*δ*_C_, type
1		150.3, C		152.3, C		154.6, C
2		112.5, C		115.4, C	6.10, s	104.9, CH
3		151.9, C		162.2, C		156.3, C
4	6.51, s	115.5, CH	6.39, s	105.9, CH	6.39, s	113.7, CH
5		136.5, C		139.5, C		139.1, C
6		124.4, C	6.21, s	111.8, CH		121.9, C
7	2.55, dd (17.0, 5.4) 2.19, m	27.2, CH_2_	2.85, dd (8.2, 5.4)	28.0, CH_2_	3.12, br d (6.8)	25.1, CH_2_
8	3.52, dt (8.0, 5.4)	68.3, CH	4.51, dd (9.3, 8.2)	89.8, CH	4.95, t (6.8)	123.2, CH
9		77.1, C		70.5, C		130.7, C
10	1.23, s	25.9, CH_3_	1.08, s	24.7, CH_3_	1.58, s	25.9, CH_3_
11	1.08, s	20.2, CH_3_	1.04	21.7, CH_3_	1.56, s	18.0, CH_3_
12	2.18, s	19.5, CH_3_	2.18, s	21.6, CH_3_	2.18, s	19.8, CH_3_
1′		159.0, C		157.9, C		159.2, C
2′	5.91, s	99.0, CH	6.29, s	101.3, CH	6.23, s	101.2, CH
3′		158.9, C		160.8, C		160.8, C
4′	6.20, s	109.8, CH	6.30, s	110.6, CH	6.45, s	109.2, CH
5′		140.3, C		140.7, C		140.5, C
6′	6.06, s	106.5, CH	6.49, d	109.7, CH	6.22,s	110.5, CH
7′	2.14, s	21.7, CH_3_	2.21, s	21.7, CH_3_	2.20, s	21.7, CH_3_
1″	3.05, br d (6.8)	25.5, CH_2_	3.68, s	55.6, CH_3_	3.67, s	55.6, CH_3_
2″	4.92, t (6.8)	122.9, CH				
3″		130.9, C				
4″	1.56, s	25.9, CH_3_				
5″	1.56, s	18.0, CH_3_				
8-OH	5.07, d (4.8)		9-OH: 4.56, s			
3′-OH	9.30, s				3-OH: 9.26, br s	

**Table 2:** MIC value of compounds **1**–**6** against plant-pathogenic fungi (μg/mL).

No	*A. alternata*	*C. heterostrophus*	*G. graminis*	*G. cingulata*	*M. hiemalis*	*T. basicola*
**1**	64	− ^a^	−	32	8	−
**2**	32	−	−	−	4	−
**3**	16	32	64	−	64	−
**4**	−	16	64	−	64	32
**5**	8	−	64	32	16	64
**6**	8	−	−	16	−	−
Pr ^b^	8	8	8	16	8	32

^a^ MIC > 64 μg/mL; ^b^ Pr, positive control, prochloraz.

## Supplementary Materials

The following are available online. Figures S1–S18, HRESIMS and NMR spectra of compounds **1**–**3**; Figure S19, ECD spectrum of compound **2**; Figure S20 and Table S1, conformational analysis of **1**.
