# Supplementary material for: Antifungal Prenylated Diphenyl Ethers from Arthrinium arundinis, an Endophytic Fungus Isolated from the Leaves of Tobacco (Nicotiana tabacum L.)
Source: Molecules. 2018 Dec 2;23(12):3179. doi: 10.3390/molecules23123179 (PMC6320909; doi:10.3390/molecules23123179)
Supplement: Supplementary file 1 [file molecules-23-03179-s001.pdf]

**Supplementary Material**

# Antifungal Prenylated Diphenyl Ethers from *Arthrinium arundinis,* an Endophytic Fungus Isolated from the Leaves of Tobacco (*Nicotiana tabacum* L.)

**Peng Zhang [1], Xin Li [2], Xiao-Long Yuan [1], Yong-Mei Du [1], Bin-Gui Wang [2,*], and Zhong-Feng Zhang [1,*]**

[1] Tobacco Research Institute, Chinese Academy of Agricultural Sciences, Qingdao, Shandong 266101, China; zhangpeng@caas.cn (P. Z.); rayrock@126.com (X.-L. Y.); duyongmei@caas.cn (Y.-M. D.)

[2] Laboratory of Marine Biology and Biotechnology, Qingdao National Laboratory for Marine Science and Technology, Key Laboratory of Experimental Marine Biology, Institute of Oceanology, Chinese Academy of Sciences, Qingdao, Shandong 266071, China

* Correspondences: wangbg@ms.qdio.ac.cn (B.-G. W.); zhangzhongfeng@caas.cn (Z.-F. Z.); Tel: +86-532-66715079 (Z.-F. Z.)

# Table of Contents

**Figure S1**. HRESIMS spectrum of compound **1**

**Figure S2**. $^1$H NMR (500 MHz, DMSO-$d_6$) spectrum of compound **1**

**Figure S3**. $^{13}$C NMR (125 MHz, DMSO-$d_6$) spectrum of compound **1**

**Figure S4**. COSY spectrum of compound **1**

**Figure S5**. HSQC spectrum of compound **1**

**Figure S6**. HMBC spectrum of compound **1**

**Figure S7**. HRESIMS spectrum of compound **2**

**Figure S8**. $^1$H NMR (500 MHz, DMSO-$d_6$) spectrum of compound **2**

**Figure S9**. $^{13}$C NMR (125 MHz, DMSO-$d_6$) and DEPT spectra of compound **2**

**Figure S10**. COSY spectrum of compound **2**

**Figure S11**. HSQC spectrum of compound **2**

**Figure S12**. HMBC spectrum of compound **2**

**Figure S13**. HRESIMS spectrum of compound **3**

**Figure S14**. $^1$H NMR (500 MHz, DMSO-$d_6$) spectrum of compound **3**

**Figure S15**. $^{13}$C NMR (125 MHz, DMSO-$d_6$) and DEPT spectra of compound **3**

**Figure S16**. COSY spectrum of compound **3**

**Figure S17**. HSQC spectrum of compound **3**

**Figure S18**. HMBC spectrum of compound **3**

**Figure S19**. ECD spectrum of compound **2**

**Figure S20.** Structure and population of the low-energy conformers (>2%) of 8*R*-**1**

**Table S1.** Cartesian coordinates of the low-energy conformers (≥2%) of **1**

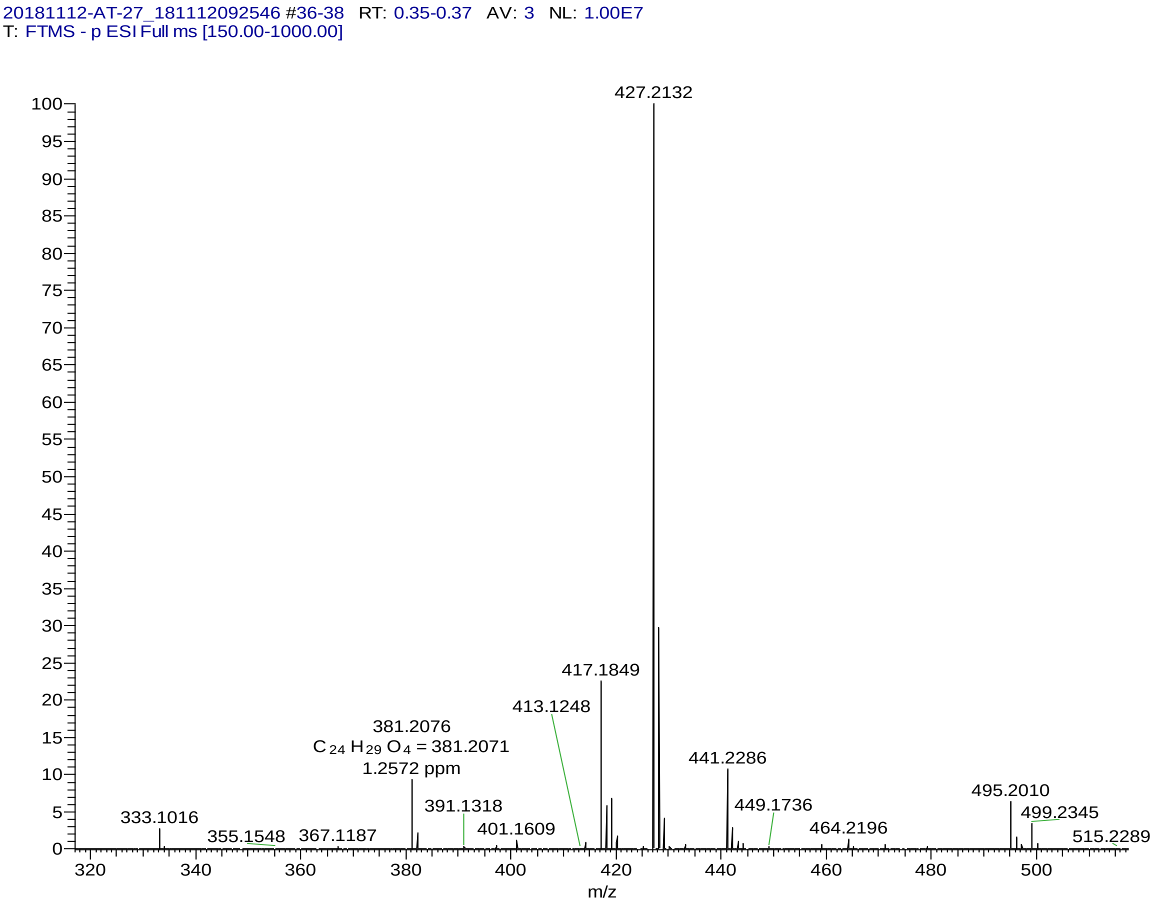


**Figure S1**. HRESIMS spectrum of compound **1**

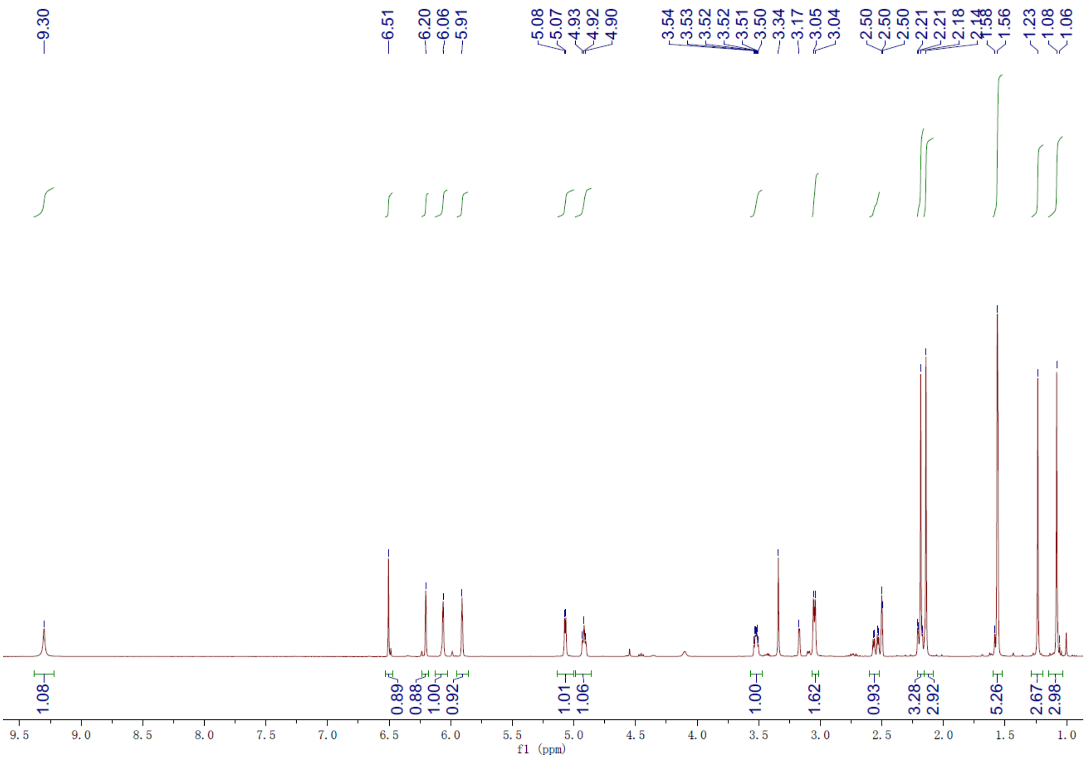


**Figure S2**. $^{1}$H NMR (500 MHz, DMSO-$d_6$) spectrum of compound **1**

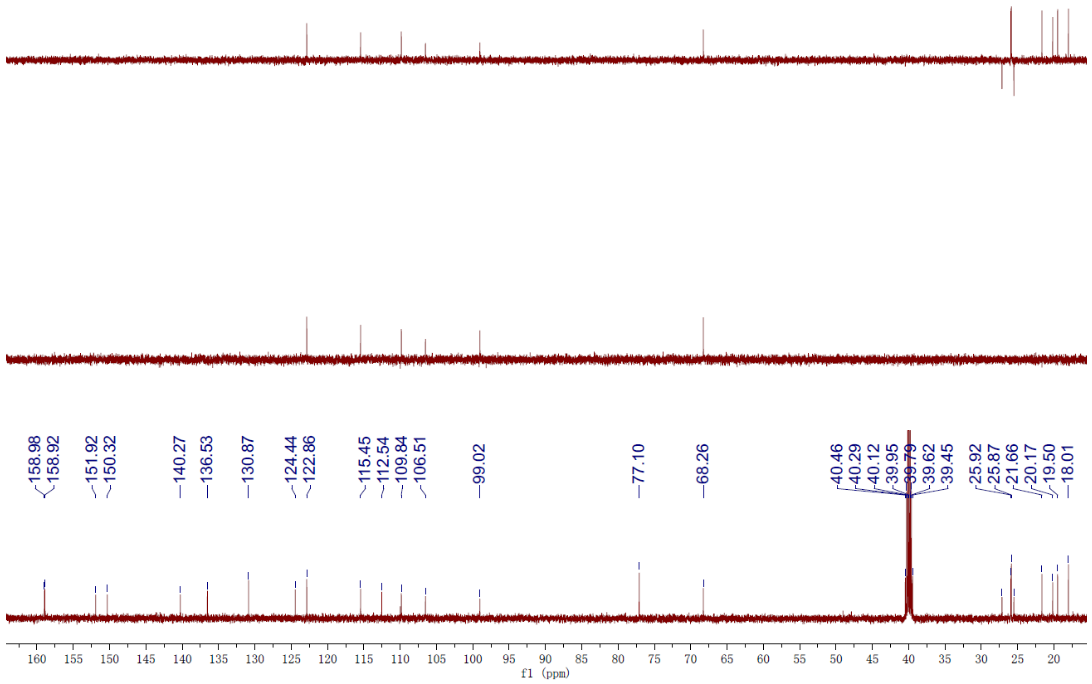


**Figure S3**. $^{13}$C NMR (125 MHz, DMSO-$d_6$) spectrum of compound **1**

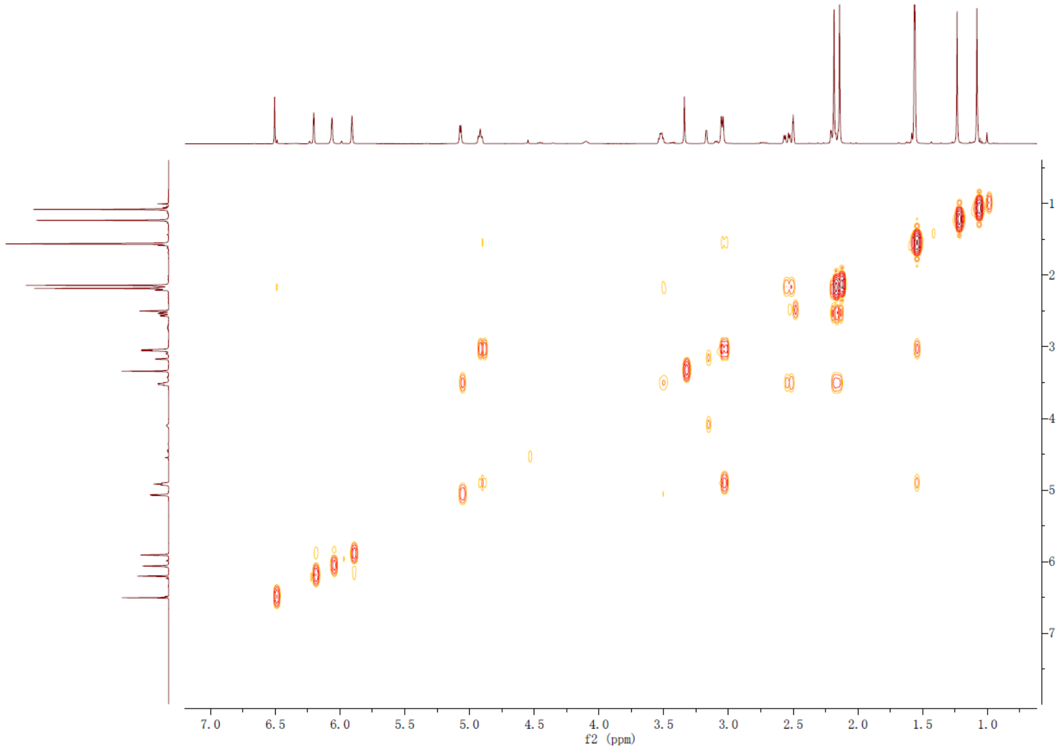


**Figure S4**. COSY spectrum of compound **1**

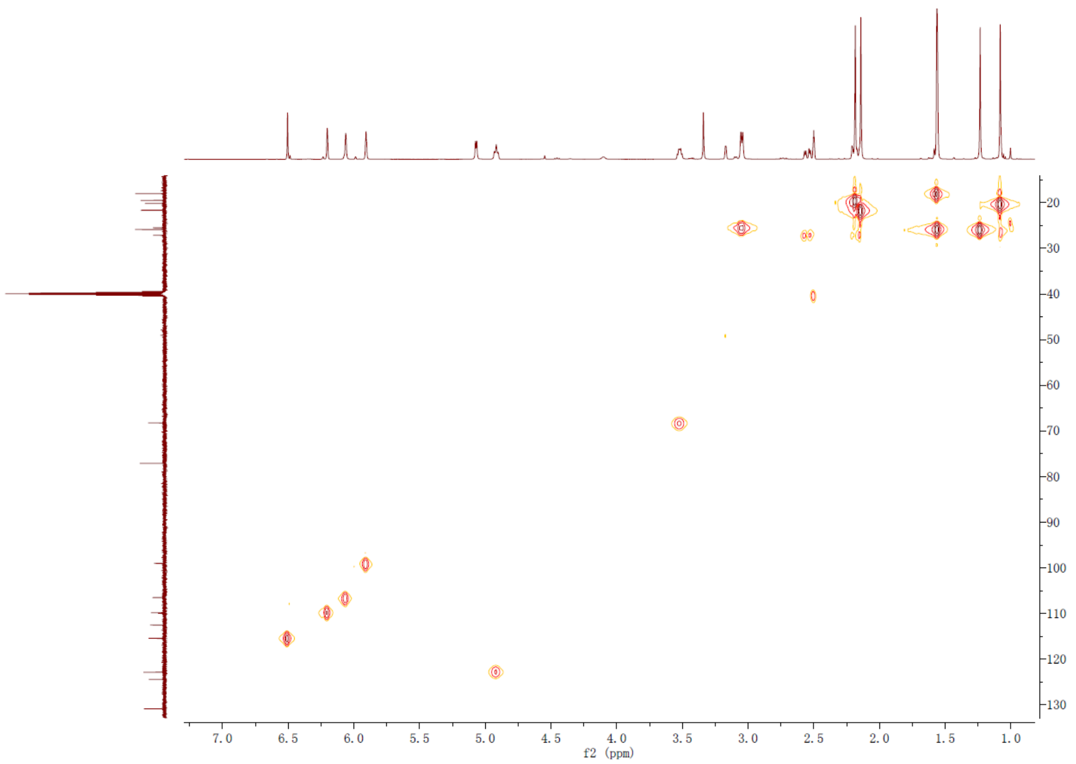


**Figure S5**. HSQC spectrum of compound **1**

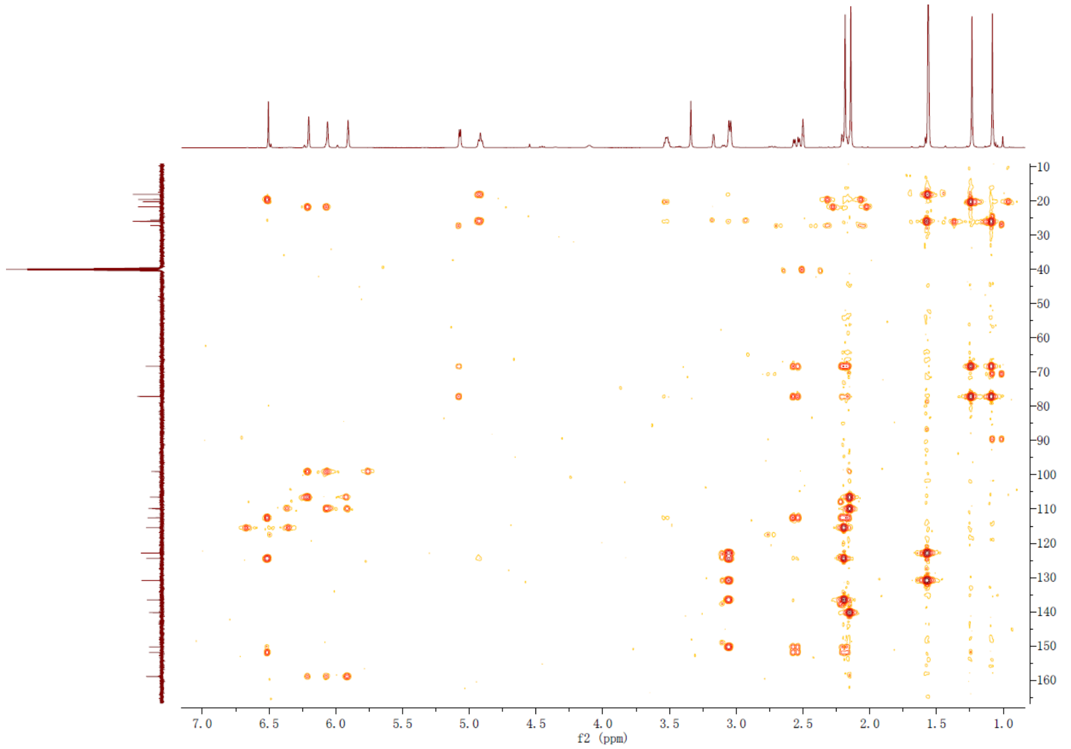


**Figure S6**. HMBC spectrum of compound **1**

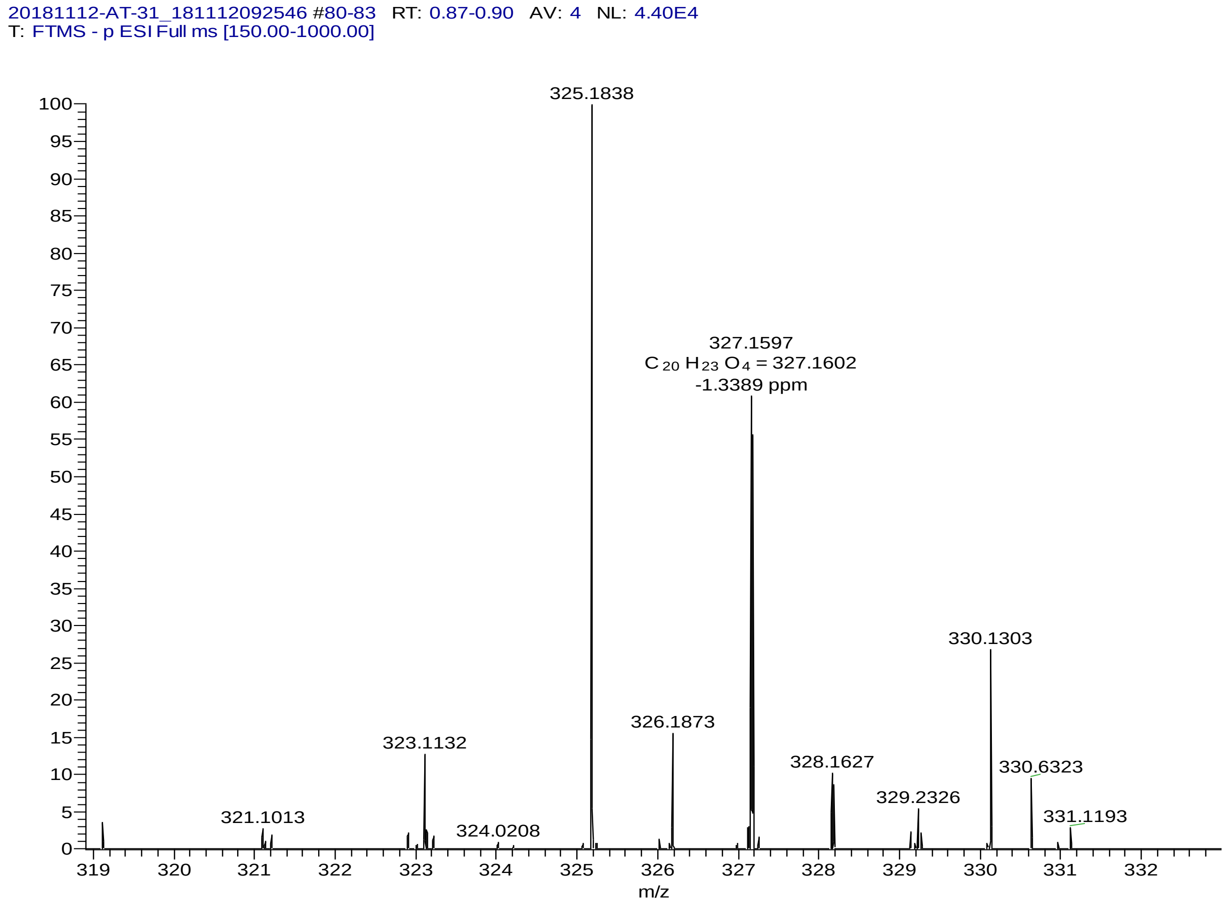


**Figure S7**. HRESIMS spectrum of compound **2**

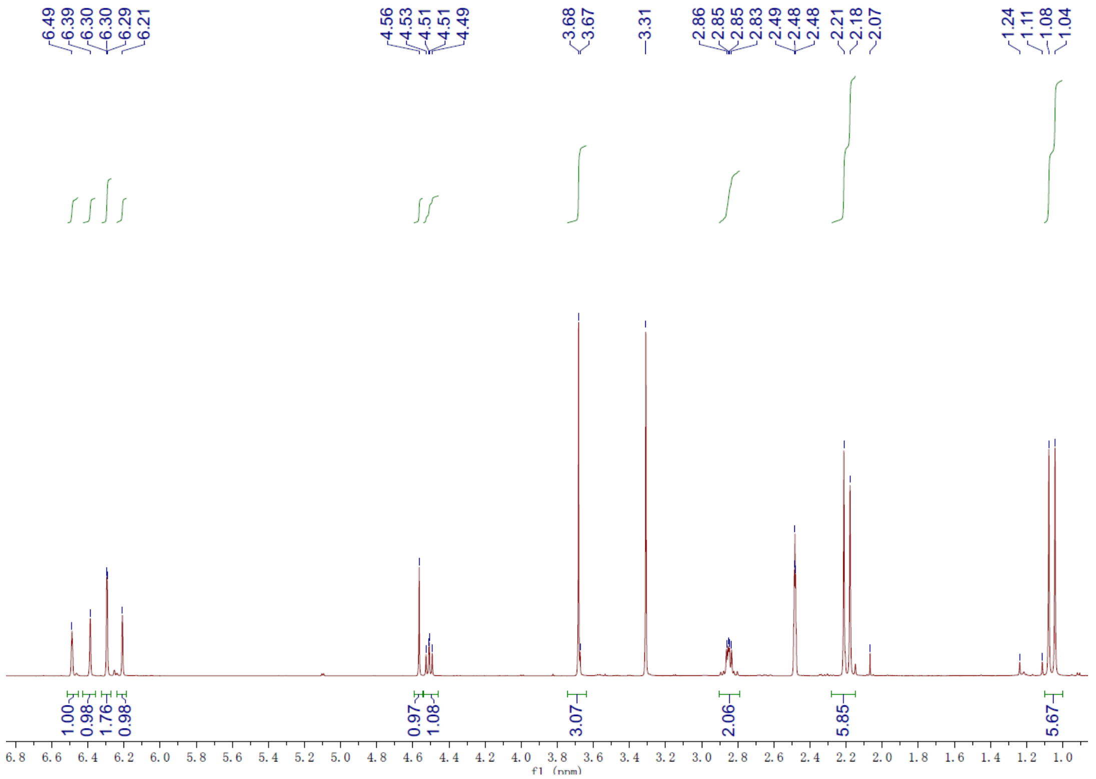


**Figure S8**. $^{1}$H NMR (500 MHz, DMSO-$d_6$) spectrum of compound **2**

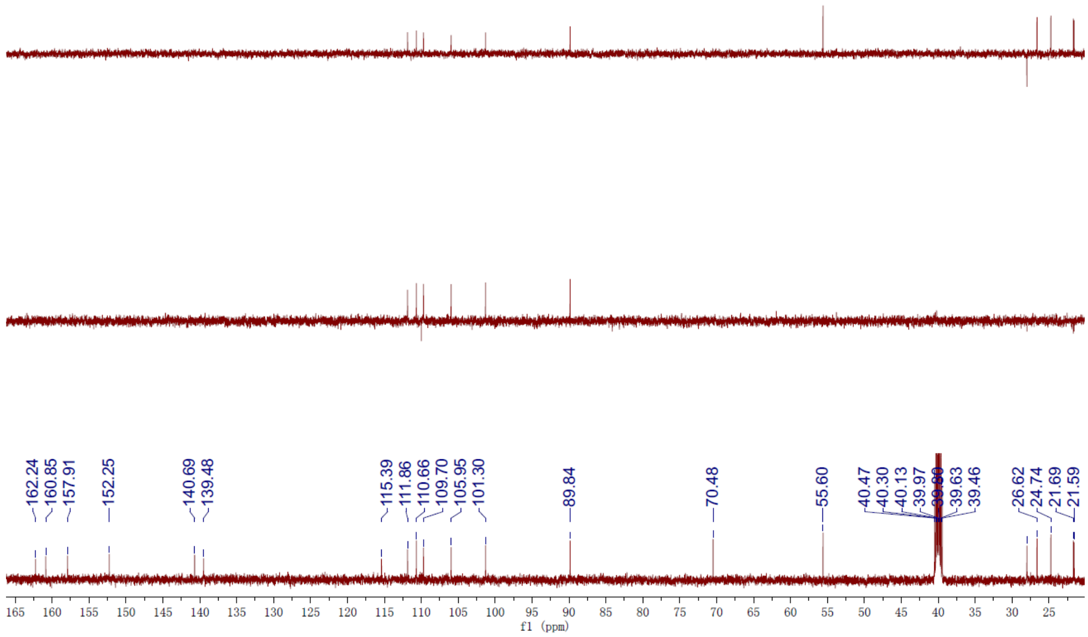


**Figure S9**. $^{13}C$ NMR (125 MHz, DMSO-$d_6$) and DEPT spectra of compound **2**

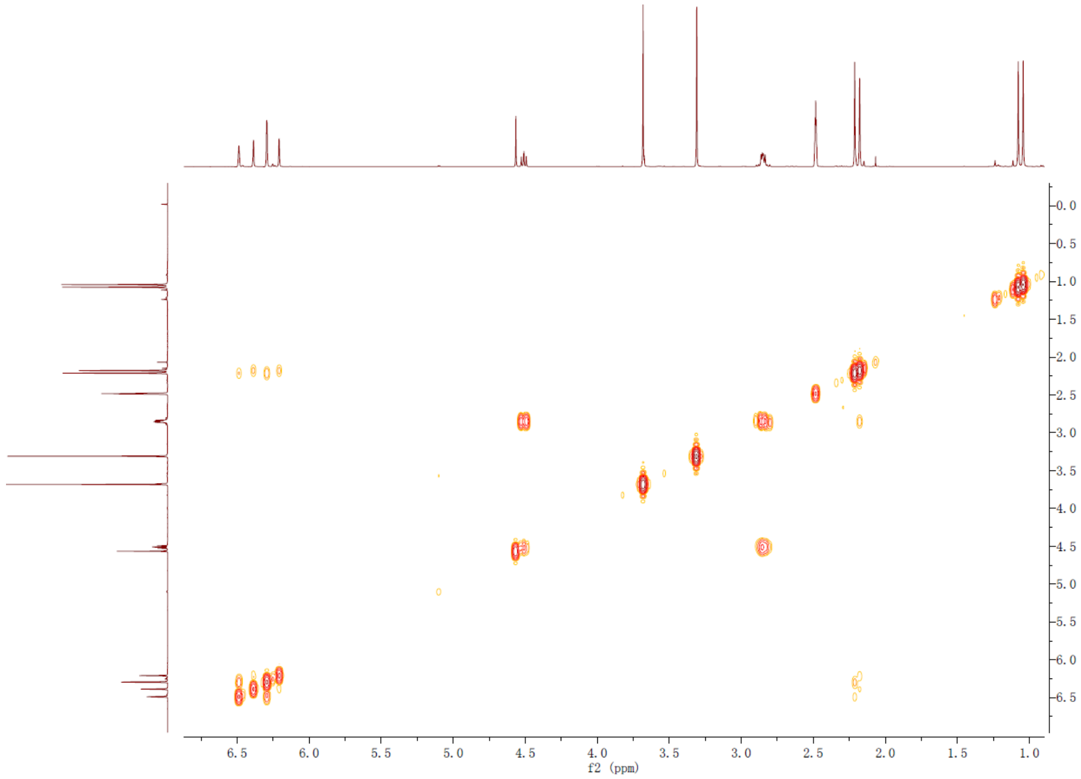


**Figure S10**. COSY spectrum of compound **2**

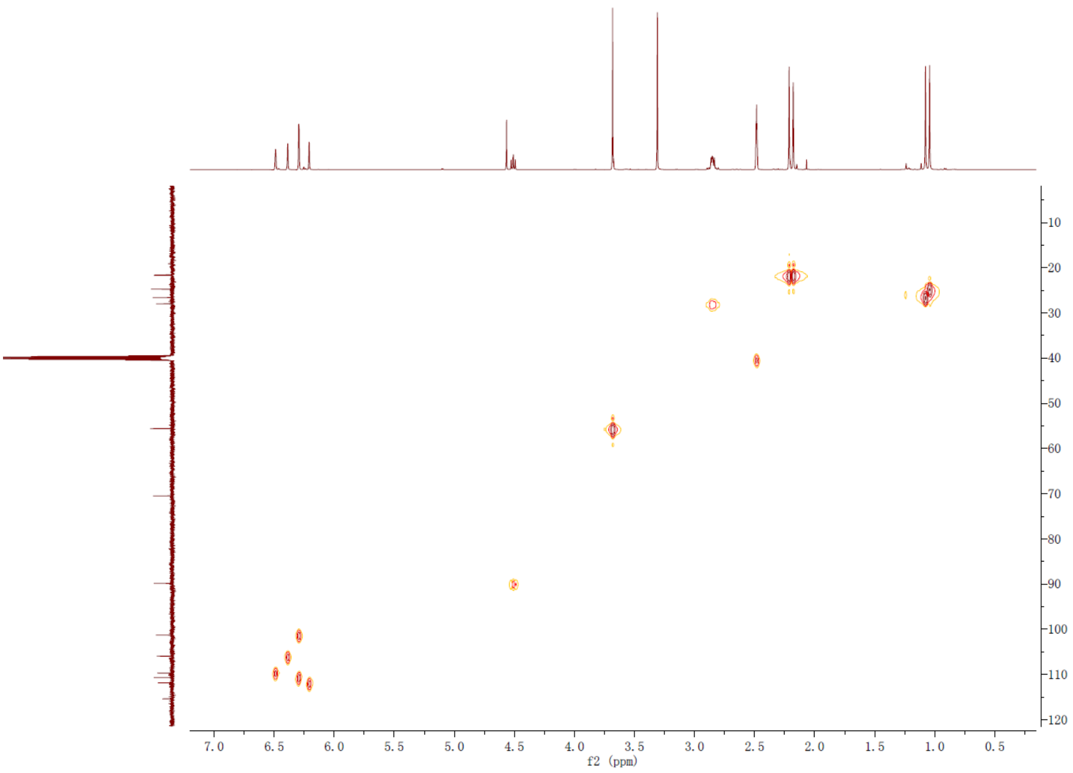


**Figure S11**. HSQC spectrum of compound **2**

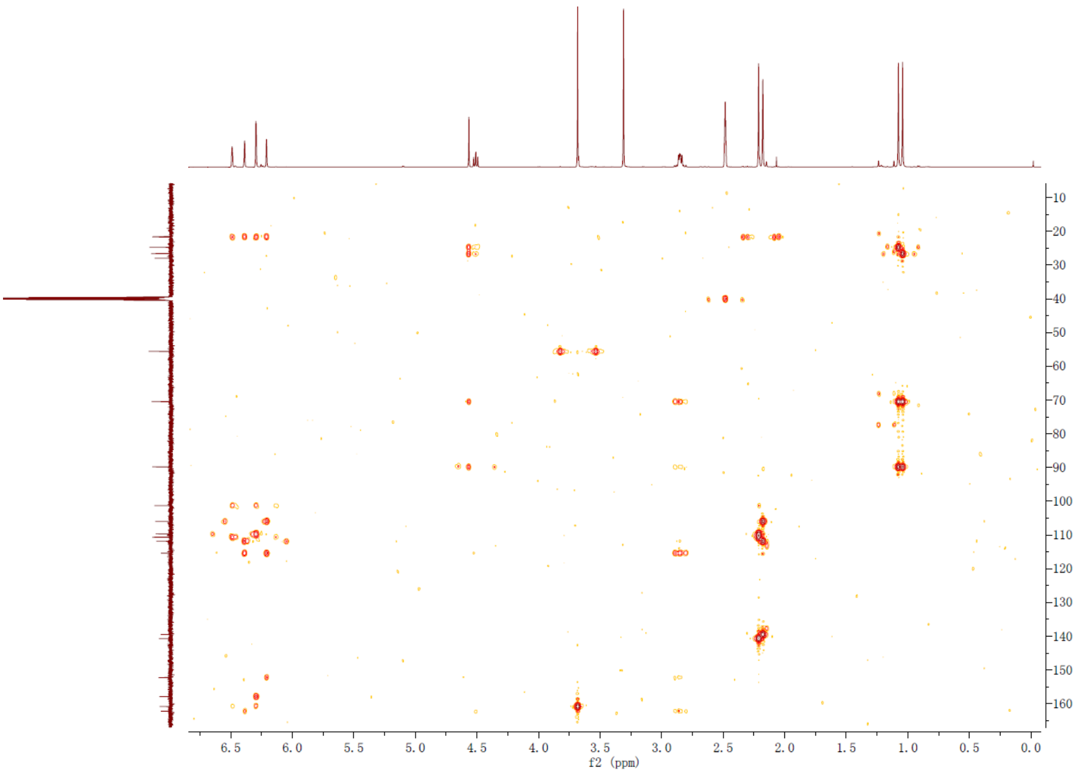


**Figure S12**. HMBC spectrum of compound **2**

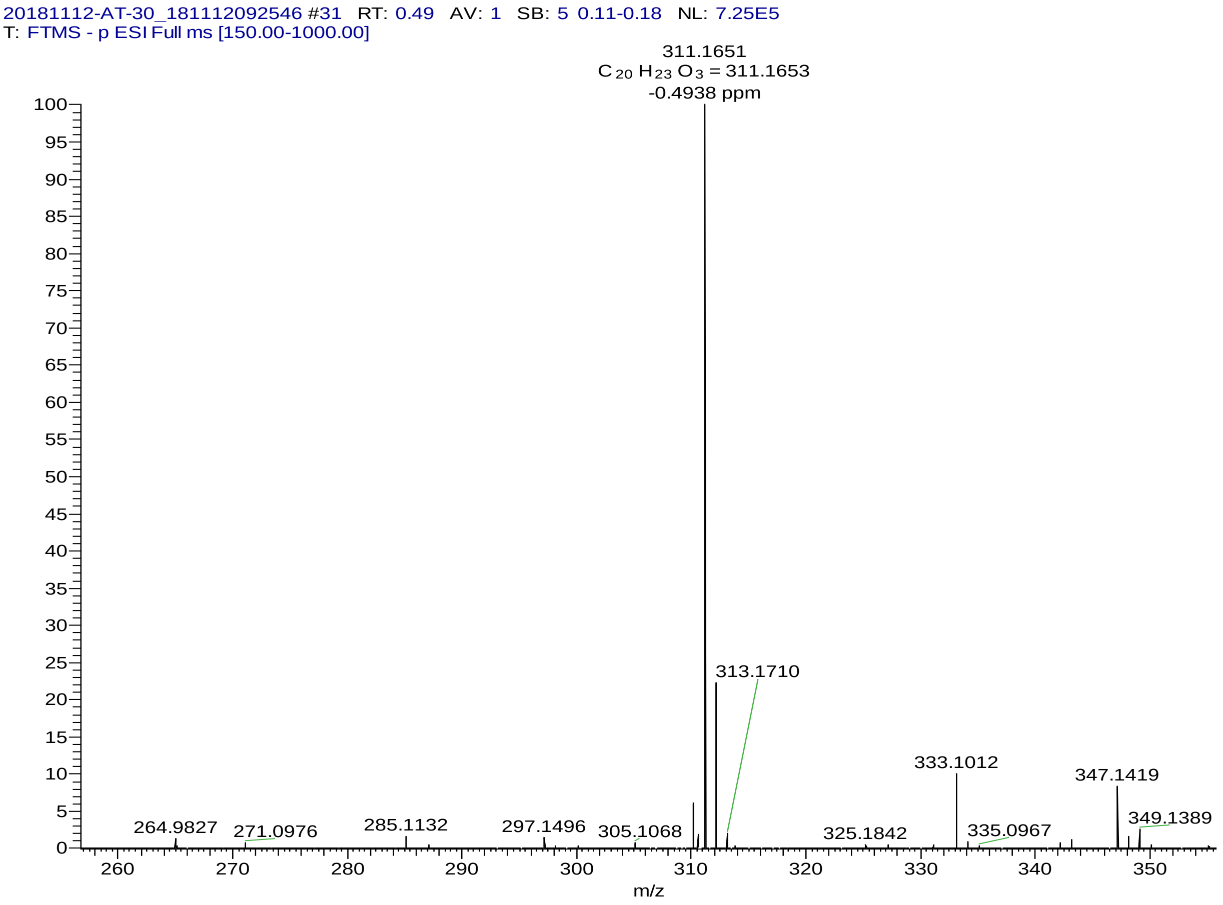


**Figure S13**. HRESIMS spectrum of compound **3**

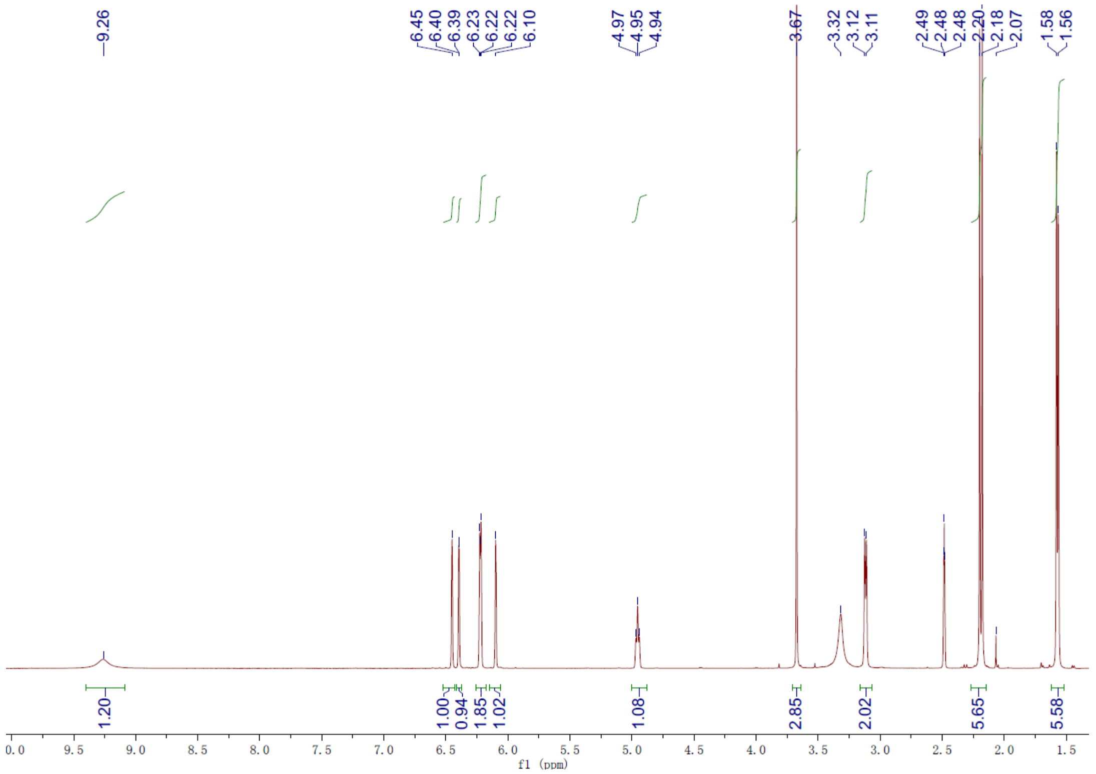


**Figure S14**. $^1$H NMR (500 MHz, DMSO-$d_6$) spectrum of compound **3**

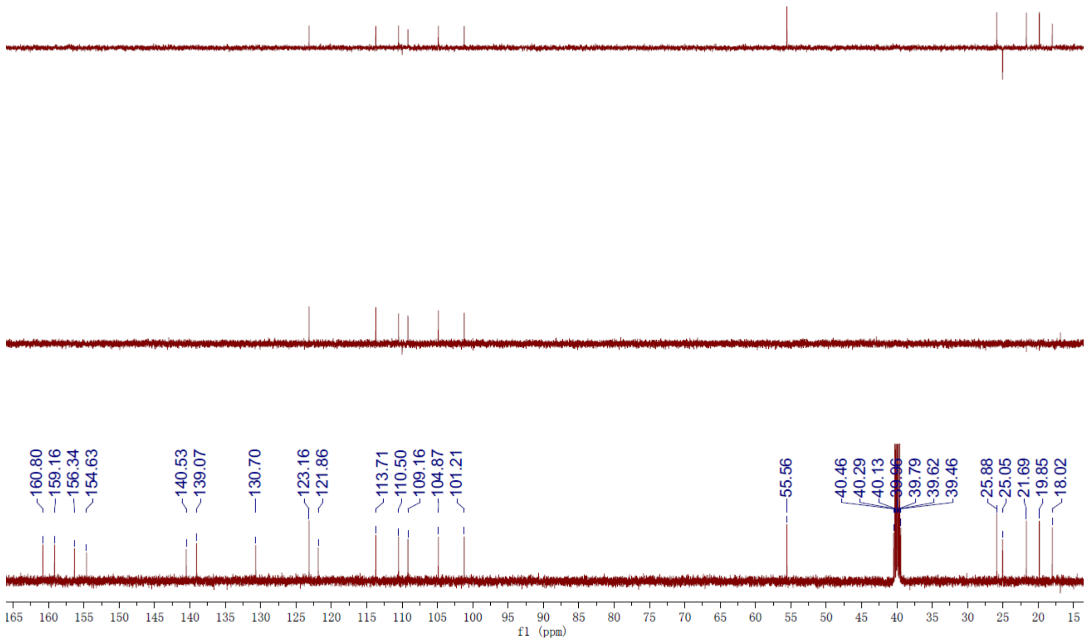

**Figure S15**. $^{13}$C NMR (125 MHz, DMSO-$d_6$) and DEPT spectra of compound **3**

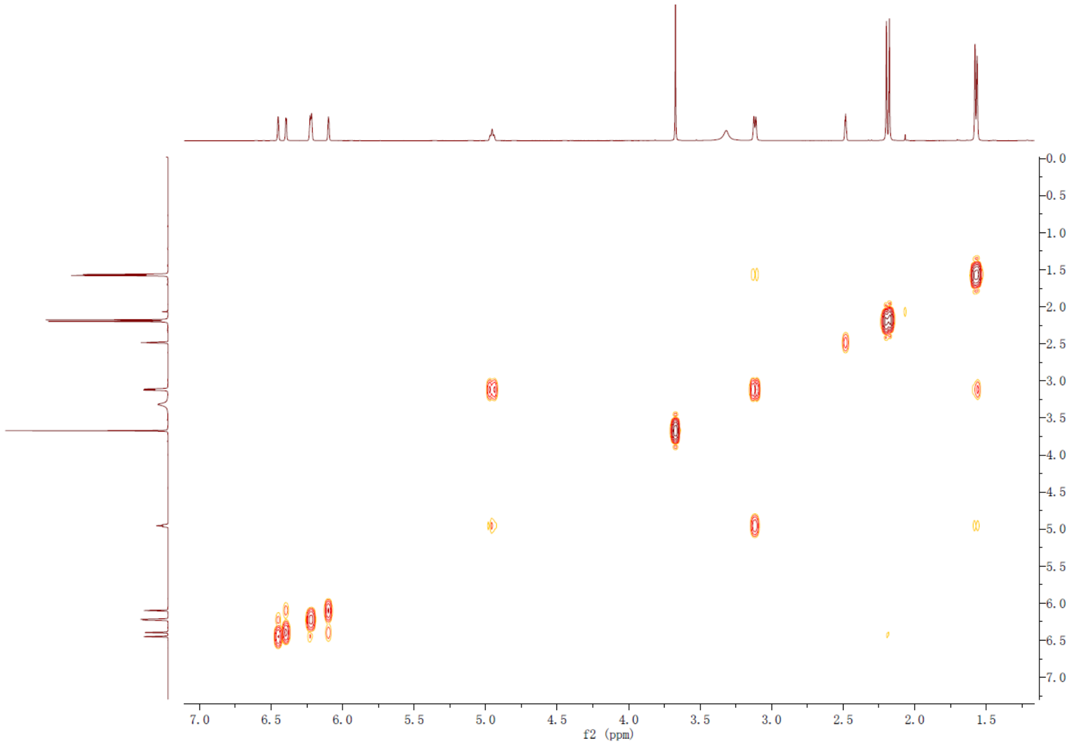

**Figure S16**. COSY spectrum of compound **3**

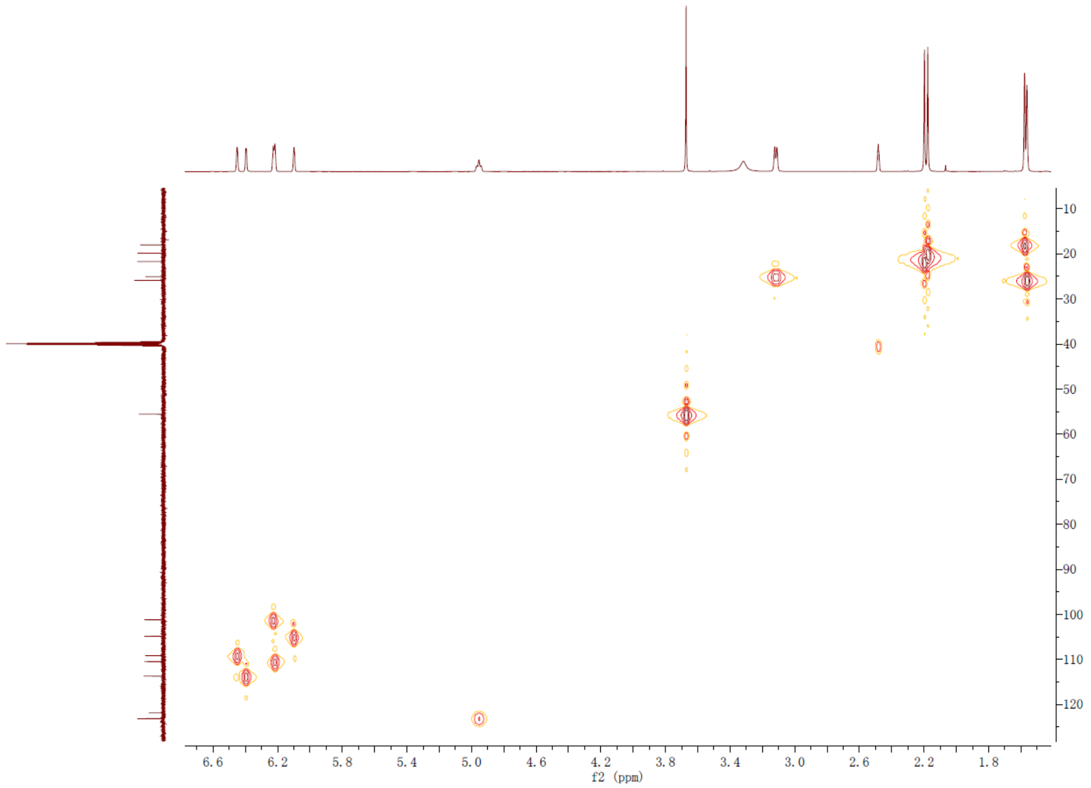


**Figure S17**. HSQC spectrum of compound **3**

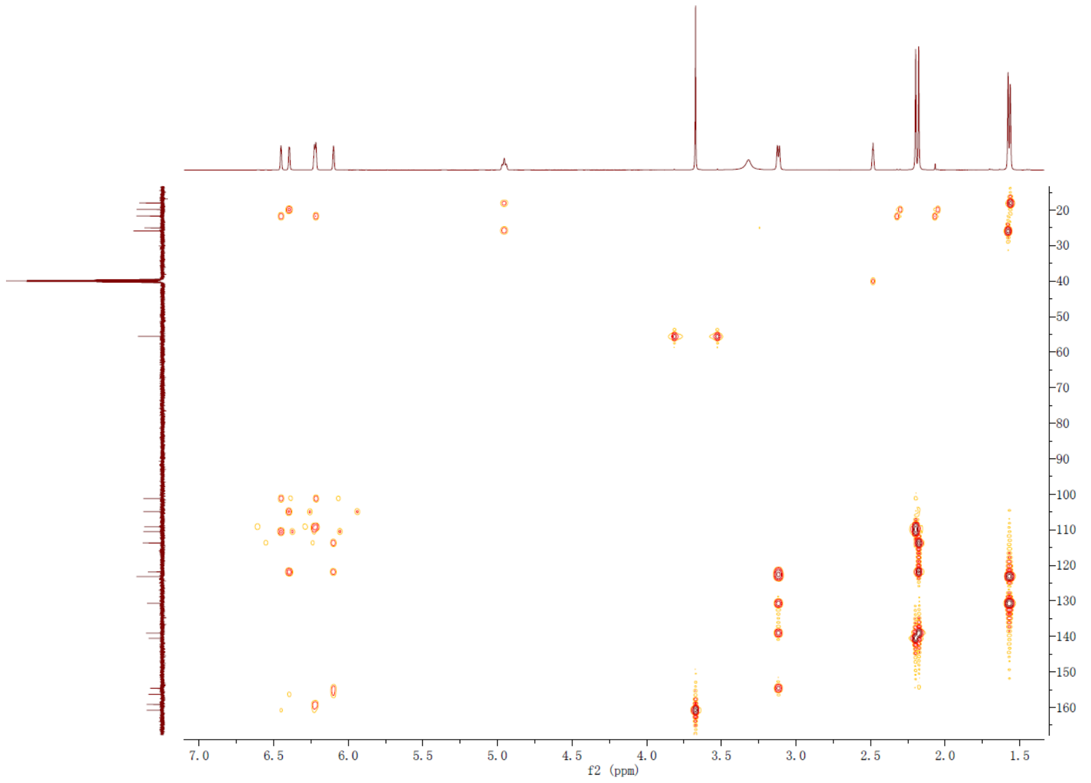


**Figure S18**. HMBC spectrum of compound **3**

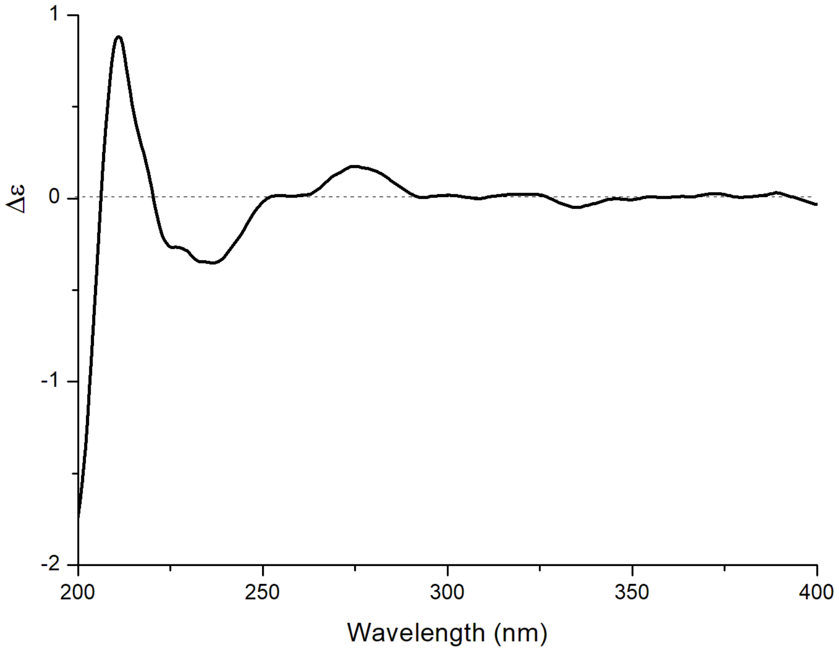


**Figure S19**. ECD spectrum of compound **2**

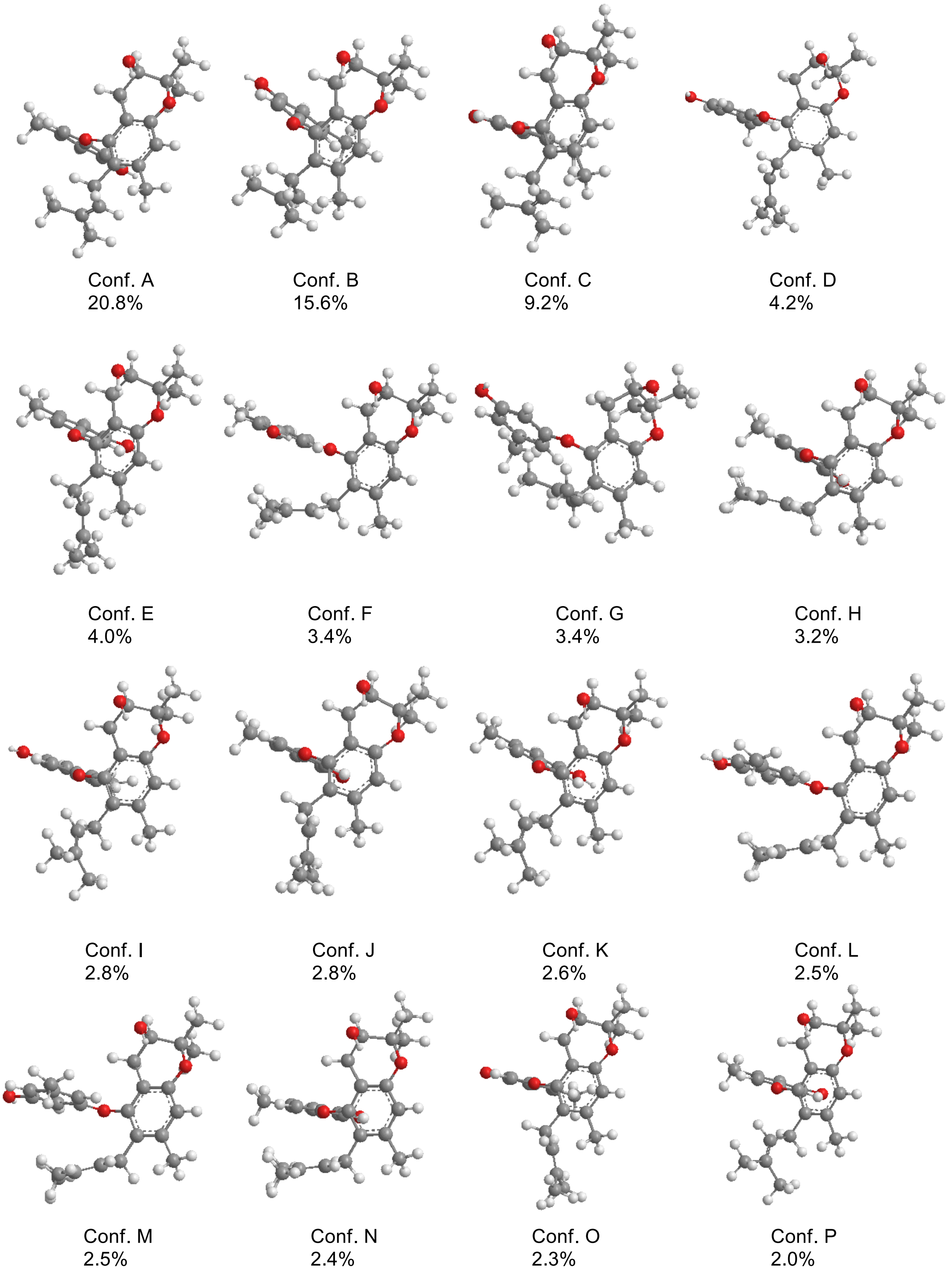


**Figure S20.** Structure and population of the low-energy conformers (>2%) of 8*R*-**1**

**Table S1.** Cartesian coordinates of the low-energy conformers (≥2%) of **1**

| (8*R*)-**1**,Conf. A | | Standard Orientation (Ångstroms) | | |
|---|---|---|---|---|
| No. | Atom | X | Y | Z |
| 1 | C | -4.255 | -2.126 | 3.725 |
| 2 | C | -4.7047 | -3.1605 | 2.8974 |
| 3 | C | -3.8038 | -3.8515 | 2.0932 |
| 4 | C | -2.4492 | -3.5336 | 2.093 |
| 5 | C | -1.9895 | -2.5061 | 2.9203 |
| 6 | C | -2.8886 | -1.8167 | 3.742 |
| 7 | O | -0.6699 | -2.1142 | 3.0347 |
| 8 | C | 0.1899 | -2.4334 | 2.0028 |
| 9 | C | 0.1638 | -1.6703 | 0.8182 |
| 10 | C | 1.0407 | -2.0137 | -0.2094 |
| 11 | C | 1.9639 | -3.0456 | -0.0374 |
| 12 | C | 2.0296 | -3.7767 | 1.1593 |
| 13 | C | 1.1371 | -3.4636 | 2.2081 |
| 14 | C | -5.2099 | -1.4042 | 4.6278 |
| 15 | O | -4.2944 | -4.8571 | 1.3116 |
| 16 | C | 1.1681 | -4.2165 | 3.5285 |
| 17 | C | 0.2954 | -5.4455 | 3.5177 |
| 18 | C | 3.078 | -4.8493 | 1.2867 |
| 19 | O | 1.1045 | -1.372 | -1.4166 |
| 20 | C | 0.0134 | -0.489 | -1.7477 |
| 21 | C | -0.4 | 0.3677 | -0.5311 |
| 22 | C | -0.787 | -0.5124 | 0.6555 |
| 23 | O | 0.6663 | 1.2436 | -0.1299 |
| 24 | C | 0.5418 | 0.4132 | -2.8752 |
| 25 | C | -1.1541 | -1.3213 | -2.3046 |
| 26 | C | -0.7023 | -5.7726 | 4.3637 |
| 27 | C | -1.1655 | -4.9464 | 5.5322 |
| 28 | C | -1.4711 | -7.0558 | 4.1808 |
| 29 | H | -5.7557 | -3.4379 | 2.8792 |
| 30 | H | -1.7596 | -4.0936 | 1.472 |
| 31 | H | -2.5145 | -1.0319 | 4.3959 |
| 32 | H | 2.6481 | -3.2718 | -0.8537 |
| 33 | H | -4.8844 | -0.3722 | 4.7952 |
| 34 | H | -6.2102 | -1.3609 | 4.1841 |
| 35 | H | -5.2726 | -1.9168 | 5.5926 |
| 36 | H | -3.5503 | -5.2767 | 0.8486 |
| 37 | H | 0.9201 | -3.5289 | 4.3419 |
| 38 | H | 2.1955 | -4.5279 | 3.7514 |

| | | | | |
|---|---|---|---|---|
| 39 | H | 0.5277 | -6.1453 | 2.7157 |
| 40 | H | 2.6255 | -5.8049 | 1.5661 |
| 41 | H | 3.605 | -5.0104 | 0.3399 |
| 42 | H | 3.8238 | -4.5619 | 2.0345 |
| 43 | H | -1.2438 | 1.0183 | -0.788 |
| 44 | H | -1.7969 | -0.9077 | 0.4998 |
| 45 | H | -0.7867 | 0.0939 | 1.5692 |
| 46 | H | 1.4565 | 0.685 | -0.0034 |
| 47 | H | -0.2085 | 1.1464 | -3.1887 |
| 48 | H | 0.8282 | -0.1835 | -3.7493 |
| 49 | H | 1.4473 | 0.9484 | -2.5679 |
| 50 | H | -1.9916 | -0.6823 | -2.603 |
| 51 | H | -0.8328 | -1.9007 | -3.1782 |
| 52 | H | -1.5232 | -2.0544 | -1.5803 |
| 53 | H | -2.2292 | -4.7083 | 5.4268 |
| 54 | H | -0.6306 | -4.0015 | 5.642 |
| 55 | H | -1.0315 | -5.5078 | 6.4629 |
| 56 | H | -2.5325 | -6.8412 | 4.0176 |
| 57 | H | -1.1149 | -7.6364 | 3.3234 |
| 58 | H | -1.3753 | -7.6854 | 5.0714 |

| (8*R*)-**1**,Conf. B | | Standard Orientation (Ångstroms) | | |
|---|---|---|---|---|
| No. | Atom | X | Y | Z |
| 1 | C | -3.6738 | -4.2476 | 1.6862 |
| 2 | C | -4.6751 | -3.6611 | 2.4683 |
| 3 | C | -4.3589 | -2.6303 | 3.3469 |
| 4 | C | -3.0525 | -2.1708 | 3.4691 |
| 5 | C | -2.044 | -2.7509 | 2.6962 |
| 6 | C | -2.353 | -3.7943 | 1.8156 |
| 7 | O | -0.7775 | -2.242 | 2.9082 |
| 8 | C | 0.1846 | -2.4784 | 1.9469 |
| 9 | C | 0.1866 | -1.7101 | 0.7656 |
| 10 | C | 1.1701 | -1.9663 | -0.1887 |
| 11 | C | 2.163 | -2.9157 | 0.0547 |
| 12 | C | 2.1951 | -3.6505 | 1.2505 |
| 13 | C | 1.1982 | -3.4248 | 2.2251 |
| 14 | C | -3.994 | -5.3938 | 0.7738 |
| 15 | O | -5.3726 | -2.0929 | 4.085 |
| 16 | C | 1.1876 | -4.1862 | 3.541 |
| 17 | C | 0.4171 | -5.4787 | 3.4577 |
| 18 | C | 3.3163 | -4.6332 | 1.4578 |

| | | | | |
|---|---|---|---|---|
| 19 | O | 1.2739 | -1.3099 | -1.3853 |
| 20 | C | 0.1361 | -0.5289 | -1.8037 |
| 21 | C | -0.449 | 0.2786 | -0.6245 |
| 22 | C | -0.8481 | -0.6409 | 0.5276 |
| 23 | O | 0.4982 | 1.2441 | -0.1387 |
| 24 | C | 0.6677 | 0.4251 | -2.8861 |
| 25 | C | -0.9023 | -1.4607 | -2.4514 |
| 26 | C | -0.602 | -5.8988 | 4.2343 |
| 27 | C | -1.1969 | -5.1366 | 5.386 |
| 28 | C | -1.2594 | -7.2304 | 3.9782 |
| 29 | H | -5.7036 | -4.007 | 2.4016 |
| 30 | H | -2.7999 | -1.3707 | 4.1571 |
| 31 | H | -1.5715 | -4.2772 | 1.2348 |
| 32 | H | 2.9267 | -3.0752 | -0.7047 |
| 33 | H | -3.8543 | -6.3421 | 1.3018 |
| 34 | H | -5.0285 | -5.3355 | 0.4193 |
| 35 | H | -3.347 | -5.3804 | -0.1096 |
| 36 | H | -5.0067 | -1.3937 | 4.6515 |
| 37 | H | 0.8239 | -3.5273 | 4.3343 |
| 38 | H | 2.216 | -4.4188 | 3.8413 |
| 39 | H | 0.748 | -6.1398 | 2.6572 |
| 40 | H | 2.9245 | -5.6253 | 1.6994 |
| 41 | H | 3.927 | -4.7423 | 0.555 |
| 42 | H | 3.9747 | -4.291 | 2.2623 |
| 43 | H | -1.3249 | 0.8523 | -0.9484 |
| 44 | H | -1.803 | -1.1238 | 0.292 |
| 45 | H | -0.9748 | -0.0435 | 1.4384 |
| 46 | H | 1.3248 | 0.7584 | 0.044 |
| 47 | H | -0.1195 | 1.0882 | -3.2597 |
| 48 | H | 1.0768 | -0.1372 | -3.7338 |
| 49 | H | 1.4925 | 1.0393 | -2.5079 |
| 50 | H | -1.768 | -0.8987 | -2.8168 |
| 51 | H | -0.4607 | -2.0031 | -3.2956 |
| 52 | H | -1.26 | -2.2286 | -1.7581 |
| 53 | H | -2.2656 | -4.9696 | 5.2149 |
| 54 | H | -0.7377 | -4.1604 | 5.551 |
| 55 | H | -1.0844 | -5.7107 | 6.3118 |
| 56 | H | -2.3221 | -7.091 | 3.7539 |
| 57 | H | -0.8095 | -7.7618 | 3.1329 |
| 58 | H | -1.1703 | -7.8731 | 4.8601 |

| (8*R*)-**1**,Conf. C | | Standard Orientation (Ångstroms) | | |
|---|---|---|---|---|
| No. | Atom | X | Y | Z |
| 1 | C | -3.6346 | -4.2169 | 1.7237 |
| 2 | C | -4.6365 | -3.6386 | 2.5129 |
| 3 | C | -4.3161 | -2.604 | 3.3882 |
| 4 | C | -3.0118 | -2.1382 | 3.4977 |
| 5 | C | -2.0042 | -2.7097 | 2.7203 |
| 6 | C | -2.3151 | -3.7547 | 1.8426 |
| 7 | O | -0.7395 | -2.194 | 2.9233 |
| 8 | C | 0.2207 | -2.4369 | 1.962 |
| 9 | C | 0.2222 | -1.6729 | 0.7781 |
| 10 | C | 1.2002 | -1.9379 | -0.1792 |
| 11 | C | 2.1901 | -2.8905 | 0.0646 |
| 12 | C | 2.2243 | -3.6195 | 1.2639 |
| 13 | C | 1.2316 | -3.3859 | 2.241 |
| 14 | C | -3.9492 | -5.366 | 0.8121 |
| 15 | O | -5.2674 | -2.0176 | 4.1706 |
| 16 | C | 1.2235 | -4.1398 | 3.5611 |
| 17 | C | 0.4547 | -5.4342 | 3.4869 |
| 18 | C | 3.3438 | -4.6038 | 1.4726 |
| 19 | O | 1.301 | -1.2882 | -1.3796 |
| 20 | C | 0.1654 | -0.5024 | -1.7949 |
| 21 | C | -0.4094 | 0.3137 | -0.6166 |
| 22 | C | -0.8074 | -0.5982 | 0.5421 |
| 23 | O | 0.5454 | 1.2774 | -0.1425 |
| 24 | C | 0.6964 | 0.4437 | -2.8846 |
| 25 | C | -0.8817 | -1.4312 | -2.4329 |
| 26 | C | -0.5623 | -5.8507 | 4.2682 |
| 27 | C | -1.1567 | -5.0804 | 5.4151 |
| 28 | C | -1.2166 | -7.1858 | 4.0239 |
| 29 | H | -5.6563 | -4.005 | 2.4407 |
| 30 | H | -2.779 | -1.3345 | 4.19 |
| 31 | H | -1.5348 | -4.2327 | 1.2561 |
| 32 | H | 2.9503 | -3.0562 | -0.697 |
| 33 | H | -4.9865 | -5.318 | 0.4646 |
| 34 | H | -3.3084 | -5.3452 | -0.0758 |
| 35 | H | -3.7962 | -6.3132 | 1.3384 |
| 36 | H | -6.1218 | -2.4438 | 3.9937 |
| 37 | H | 0.8607 | -3.4769 | 4.3516 |
| 38 | H | 2.2528 | -4.3693 | 3.8608 |
| 39 | H | 0.7861 | -6.1001 | 2.6908 |
| 40 | H | 4.0058 | -4.259 | 2.273 |

| | | | | |
|---|---|---|---|---|
| 41 | H | 2.9507 | -5.5938 | 1.7206 |
| 42 | H | 3.9512 | -4.719 | 0.5683 |
| 43 | H | -1.2843 | 0.89 | -0.9388 |
| 44 | H | -1.7666 | -1.0762 | 0.3146 |
| 45 | H | -0.9247 | 0.0037 | 1.4512 |
| 46 | H | 1.3646 | 0.7861 | 0.0574 |
| 47 | H | -0.089 | 1.1096 | -3.2568 |
| 48 | H | 1.0976 | -0.1248 | -3.732 |
| 49 | H | 1.5268 | 1.0549 | -2.5137 |
| 50 | H | -1.7463 | -0.8659 | -2.7962 |
| 51 | H | -0.4478 | -1.9799 | -3.2771 |
| 52 | H | -1.24 | -2.194 | -1.7344 |
| 53 | H | -0.7018 | -4.1006 | 5.5696 |
| 54 | H | -1.0382 | -5.6456 | 6.3457 |
| 55 | H | -2.2268 | -4.9203 | 5.2463 |
| 56 | H | -2.2801 | -7.0509 | 3.8008 |
| 57 | H | -0.7672 | -7.7226 | 3.1818 |
| 58 | H | -1.124 | -7.8213 | 4.9106 |

| (8*R*)-**1**,Conf. D | | Standard Orientation (Ångstroms) | | |
|---|---|---|---|---|
| No. | Atom | X | Y | Z |
| 1 | C | -3.9847 | -3.9304 | 1.5029 |
| 2 | C | -4.9951 | -3.4085 | 2.3182 |
| 3 | C | -4.6942 | -2.4309 | 3.2604 |
| 4 | C | -3.3939 | -1.9629 | 3.4144 |
| 5 | C | -2.374 | -2.4823 | 2.6132 |
| 6 | C | -2.6695 | -3.4704 | 1.6656 |
| 7 | O | -1.1202 | -1.9543 | 2.8567 |
| 8 | C | -0.0886 | -2.3108 | 2.0131 |
| 9 | C | 0.0681 | -1.6181 | 0.7963 |
| 10 | C | 1.0992 | -2.0051 | -0.0573 |
| 11 | C | 1.9908 | -3.0089 | 0.3212 |
| 12 | C | 1.8777 | -3.6592 | 1.5609 |
| 13 | C | 0.8251 | -3.3048 | 2.4332 |
| 14 | C | -4.2912 | -5.0139 | 0.512 |
| 15 | O | -5.7168 | -1.9529 | 4.0264 |
| 16 | C | 0.6682 | -3.9663 | 3.7933 |
| 17 | C | 0.0012 | -5.3146 | 3.7207 |
| 18 | C | 2.8797 | -4.7294 | 1.9014 |
| 19 | O | 1.3434 | -1.4375 | -1.2783 |
| 20 | C | 0.3036 | -0.606 | -1.8327 |

| | | | | |
|---|---|---|---|---|
| 21 | C | -0.3079 | 0.3156 | -0.7545 |
| 22 | C | -0.8624 | -0.4965 | 0.414 |
| 23 | O | 0.6694 | 1.2413 | -0.2515 |
| 24 | C | 0.9873 | 0.236 | -2.9227 |
| 25 | C | -0.7499 | -1.5017 | -2.5066 |
| 26 | C | 0.4452 | -6.4991 | 4.1871 |
| 27 | C | 1.7472 | -6.7247 | 4.9039 |
| 28 | C | -0.385 | -7.7453 | 4.0136 |
| 29 | H | -6.0193 | -3.7612 | 2.2257 |
| 30 | H | -3.1555 | -1.2011 | 4.1497 |
| 31 | H | -1.8859 | -3.9 | 1.0466 |
| 32 | H | 2.7912 | -3.2795 | -0.3655 |
| 33 | H | -4.1424 | -5.9951 | 0.9731 |
| 34 | H | -5.3256 | -4.942 | 0.1598 |
| 35 | H | -3.6429 | -4.9322 | -0.3667 |
| 36 | H | -5.3606 | -1.2913 | 4.6422 |
| 37 | H | 0.0688 | -3.3308 | 4.4564 |
| 38 | H | 1.6468 | -4.0025 | 4.2791 |
| 39 | H | -0.9747 | -5.2948 | 3.2368 |
| 40 | H | 2.3968 | -5.7104 | 1.9194 |
| 41 | H | 3.6849 | -4.7739 | 1.1598 |
| 42 | H | 3.3516 | -4.5285 | 2.8676 |
| 43 | H | -1.1106 | 0.9274 | -1.1818 |
| 44 | H | -1.8307 | -0.9251 | 0.1327 |
| 45 | H | -1.0123 | 0.1653 | 1.2754 |
| 46 | H | 1.4415 | 0.712 | 0.0247 |
| 47 | H | 0.2837 | 0.928 | -3.397 |
| 48 | H | 1.416 | -0.4076 | -3.7 |
| 49 | H | 1.8251 | 0.813 | -2.5156 |
| 50 | H | -1.54 | -0.9044 | -2.9735 |
| 51 | H | -0.2874 | -2.1276 | -3.2788 |
| 52 | H | -1.217 | -2.1958 | -1.8006 |
| 53 | H | 1.5615 | -7.1632 | 5.8902 |
| 54 | H | 2.3248 | -5.8122 | 5.061 |
| 55 | H | 2.3748 | -7.4167 | 4.3324 |
| 56 | H | -0.6351 | -8.1732 | 4.9899 |
| 57 | H | -1.3245 | -7.5506 | 3.4857 |
| 58 | H | 0.1704 | -8.4926 | 3.4373 |

| (8*R*)-**1**,Conf. E | | Standard Orientation (Ångstroms) | | |
|---|---|---|---|---|
| No. | Atom | X | Y | Z |

| | | | | |
|---|---|---|---|---|
| 1 | C | -4.5784 | -1.8931 | 3.6624 |
| 2 | C | -5.0098 | -2.8887 | 2.7795 |
| 3 | C | -4.095 | -3.5268 | 1.9481 |
| 4 | C | -2.7431 | -3.1973 | 1.9788 |
| 5 | C | -2.2995 | -2.2102 | 2.8638 |
| 6 | C | -3.2155 | -1.5706 | 3.7071 |
| 7 | O | -0.9894 | -1.7933 | 3.0068 |
| 8 | C | -0.0612 | -2.2445 | 2.0916 |
| 9 | C | 0.0474 | -1.5901 | 0.8485 |
| 10 | C | 0.9659 | -2.0785 | -0.0783 |
| 11 | C | 1.8003 | -3.1471 | 0.2499 |
| 12 | C | 1.7421 | -3.7611 | 1.5118 |
| 13 | C | 0.8025 | -3.3016 | 2.4606 |
| 14 | C | -5.5493 | -1.2267 | 4.5905 |
| 15 | O | -4.5702 | -4.4898 | 1.1058 |
| 16 | C | 0.7145 | -3.9149 | 3.8494 |
| 17 | C | -0.0778 | -5.1952 | 3.882 |
| 18 | C | 2.6809 | -4.9024 | 1.796 |
| 19 | O | 1.1512 | -1.5564 | -1.3295 |
| 20 | C | 0.1466 | -0.6418 | -1.8136 |
| 21 | C | -0.2903 | 0.3453 | -0.7089 |
| 22 | C | -0.8139 | -0.3985 | 0.5181 |
| 23 | O | 0.8017 | 1.1887 | -0.3081 |
| 24 | C | 0.8089 | 0.1208 | -2.973 |
| 25 | C | -1.0338 | -1.4489 | -2.3804 |
| 26 | C | 0.3032 | -6.4101 | 4.3257 |
| 27 | C | 1.6509 | -6.7494 | 4.8991 |
| 28 | C | -0.6546 | -7.5732 | 4.2785 |
| 29 | H | -6.058 | -3.1743 | 2.7366 |
| 30 | H | -2.0476 | -3.7132 | 1.3265 |
| 31 | H | -2.8578 | -0.813 | 4.4015 |
| 32 | H | 2.5145 | -3.4965 | -0.4941 |
| 33 | H | -5.2361 | -0.201 | 4.8121 |
| 34 | H | -6.5458 | -1.1713 | 4.1398 |
| 35 | H | -5.6155 | -1.7871 | 5.5281 |
| 36 | H | -3.8248 | -4.8641 | 0.6077 |
| 37 | H | 0.2417 | -3.2098 | 4.544 |
| 38 | H | 1.7266 | -4.0324 | 4.2456 |
| 39 | H | -1.0971 | -5.0885 | 3.5133 |
| 40 | H | 2.123 | -5.8375 | 1.8968 |
| 41 | H | 3.4033 | -5.0384 | 0.9836 |
| 42 | H | 3.2595 | -4.7134 | 2.705 |
| 43 | H | -1.0702 | 1.019 | -1.0821 |

| | | | | |
|---|---|---|---|---|
| 44 | H | -1.8347 | -0.7463 | 0.3254 |
| 45 | H | -0.8357 | 0.2866 | 1.374 |
| 46 | H | 1.5441 | 0.5984 | -0.0782 |
| 47 | H | 0.132 | 0.8665 | -3.4026 |
| 48 | H | 1.1132 | -0.5689 | -3.7692 |
| 49 | H | 1.7252 | 0.6259 | -2.6477 |
| 50 | H | -1.8049 | -0.79 | -2.7929 |
| 51 | H | -0.694 | -2.1235 | -3.175 |
| 52 | H | -1.4989 | -2.0895 | -1.6243 |
| 53 | H | 1.5377 | -7.1499 | 5.9121 |
| 54 | H | 2.3272 | -5.8953 | 4.9641 |
| 55 | H | 2.1405 | -7.5096 | 4.2814 |
| 56 | H | -0.8308 | -7.9604 | 5.2875 |
| 57 | H | -1.6258 | -7.297 | 3.8544 |
| 58 | H | -0.2412 | -8.3789 | 3.663 |

| (8*R*)-**1**,Conf. F | | Standard Orientation (Ångstroms) | | |
|---|---|---|---|---|
| No. | Atom | X | Y | Z |
| 1 | C | -4.6707 | -3.4038 | 2.9454 |
| 2 | C | -4.2041 | -2.5644 | 3.9618 |
| 3 | C | -2.8847 | -2.1231 | 3.9498 |
| 4 | C | -2.0044 | -2.5092 | 2.9426 |
| 5 | C | -2.4543 | -3.3638 | 1.9334 |
| 6 | C | -3.7824 | -3.8083 | 1.9402 |
| 7 | O | -1.6913 | -3.8436 | 0.8862 |
| 8 | C | -0.3822 | -3.4223 | 0.7783 |
| 9 | C | -0.1052 | -2.2692 | 0.0172 |
| 10 | C | 1.227 | -1.8979 | -0.1608 |
| 11 | C | 2.2538 | -2.6335 | 0.4309 |
| 12 | C | 1.9861 | -3.7908 | 1.1788 |
| 13 | C | 0.6477 | -4.2149 | 1.3383 |
| 14 | C | -6.0797 | -3.915 | 2.9677 |
| 15 | O | -2.4837 | -1.3037 | 4.9653 |
| 16 | C | 0.3085 | -5.4884 | 2.0982 |
| 17 | C | 0.1451 | -5.2586 | 3.5789 |
| 18 | C | 3.1498 | -4.5659 | 1.7371 |
| 19 | O | 1.6339 | -0.8135 | -0.8904 |
| 20 | C | 0.6717 | -0.2483 | -1.8044 |
| 21 | C | -0.7147 | -0.1254 | -1.1374 |
| 22 | C | -1.2113 | -1.4802 | -0.6352 |
| 23 | O | -0.6683 | 0.7866 | -0.0269 |

| | | | | |
|---|---|---|---|---|
| 24 | C | 1.2139 | 1.1458 | -2.1617 |
| 25 | C | 0.6349 | -1.0924 | -3.0896 |
| 26 | C | -0.8446 | -5.691 | 4.386 |
| 27 | C | -2.0131 | -6.5366 | 3.9612 |
| 28 | C | -0.8516 | -5.3299 | 5.8492 |
| 29 | H | -4.863 | -2.2495 | 4.767 |
| 30 | H | -0.9791 | -2.1595 | 2.9588 |
| 31 | H | -4.1202 | -4.4796 | 1.1536 |
| 32 | H | 3.2814 | -2.3069 | 0.2793 |
| 33 | H | -6.4481 | -4.0883 | 1.9511 |
| 34 | H | -6.1277 | -4.8546 | 3.5266 |
| 35 | H | -6.7518 | -3.189 | 3.437 |
| 36 | H | -1.5529 | -1.0672 | 4.8193 |
| 37 | H | 1.106 | -6.2258 | 1.9491 |
| 38 | H | -0.5735 | -5.9492 | 1.6446 |
| 39 | H | 0.9365 | -4.6599 | 4.0284 |
| 40 | H | 4.0892 | -4.013 | 1.6276 |
| 41 | H | 3.0171 | -4.7558 | 2.8058 |
| 42 | H | 3.2598 | -5.5162 | 1.2054 |
| 43 | H | -1.4462 | 0.29 | -1.8399 |
| 44 | H | -1.6025 | -2.0685 | -1.4734 |
| 45 | H | -2.026 | -1.3125 | 0.0768 |
| 46 | H | 0.0804 | 0.5072 | 0.5325 |
| 47 | H | 0.5367 | 1.68 | -2.8362 |
| 48 | H | 2.1947 | 1.0695 | -2.6459 |
| 49 | H | 1.3692 | 1.7569 | -1.2655 |
| 50 | H | -0.0563 | -0.6673 | -3.8248 |
| 51 | H | 1.6314 | -1.1482 | -3.5434 |
| 52 | H | 0.3358 | -2.1277 | -2.898 |
| 53 | H | -2.0155 | -7.4778 | 4.5213 |
| 54 | H | -2.0069 | -6.7926 | 2.9003 |
| 55 | H | -2.9523 | -6.0138 | 4.1687 |
| 56 | H | -0.8198 | -6.2356 | 6.4635 |
| 57 | H | 0.0051 | -4.7079 | 6.1291 |
| 58 | H | -1.7608 | -4.772 | 6.0969 |

| (8*R*)-**1**,Conf. G | | Standard Orientation (Ångstroms) | | |
|---|---|---|---|---|
| No. | Atom | X | Y | Z |
| 1 | C | -4.1737 | -4.0593 | 0.9126 |
| 2 | C | -5.1095 | -3.6344 | 1.8628 |
| 3 | C | -4.6854 | -2.9817 | 3.0154 |

| | | | | |
|---|---|---|---|---|
| 4 | C | -3.3351 | -2.7462 | 3.2468 |
| 5 | C | -2.3914 | -3.1695 | 2.3079 |
| 6 | C | -2.8093 | -3.8368 | 1.1501 |
| 7 | O | -1.0781 | -2.9005 | 2.6406 |
| 8 | C | -0.1256 | -2.9945 | 1.6468 |
| 9 | C | 0.0497 | -1.9166 | 0.7579 |
| 10 | C | 1.0172 | -2.0324 | -0.2387 |
| 11 | C | 1.8345 | -3.1615 | -0.3044 |
| 12 | C | 1.7023 | -4.2147 | 0.6146 |
| 13 | C | 0.7018 | -4.1406 | 1.6072 |
| 14 | C | -4.6196 | -4.8004 | -0.3129 |
| 15 | O | -5.6385 | -2.5859 | 3.9073 |
| 16 | C | 0.5242 | -5.2364 | 2.6441 |
| 17 | C | 1.2474 | -4.9275 | 3.9289 |
| 18 | C | 2.6286 | -5.3944 | 0.4938 |
| 19 | O | 1.2717 | -1.073 | -1.181 |
| 20 | C | 0.291 | -0.0261 | -1.3297 |
| 21 | C | -0.2057 | 0.4735 | 0.0449 |
| 22 | C | -0.7908 | -0.6715 | 0.8689 |
| 23 | O | 0.862 | 1.0814 | 0.7901 |
| 24 | C | 1.0112 | 1.1088 | -2.0765 |
| 25 | C | -0.856 | -0.5354 | -2.2191 |
| 26 | C | 0.7332 | -4.8017 | 5.1689 |
| 27 | C | -0.7182 | -4.9539 | 5.5316 |
| 28 | C | 1.6273 | -4.4727 | 6.3364 |
| 29 | H | -6.1715 | -3.8118 | 1.7119 |
| 30 | H | -2.9983 | -2.2396 | 4.1455 |
| 31 | H | -2.0847 | -4.1946 | 0.4235 |
| 32 | H | 2.5924 | -3.2096 | -1.0846 |
| 33 | H | -3.9485 | -4.6006 | -1.1549 |
| 34 | H | -4.6305 | -5.8767 | -0.1157 |
| 35 | H | -5.6235 | -4.486 | -0.6168 |
| 36 | H | -5.2005 | -2.148 | 4.6558 |
| 37 | H | 0.9052 | -6.1894 | 2.2599 |
| 38 | H | -0.5426 | -5.4236 | 2.791 |
| 39 | H | 2.3219 | -4.7917 | 3.8053 |
| 40 | H | 3.4003 | -5.2242 | -0.2649 |
| 41 | H | 3.1452 | -5.5778 | 1.4409 |
| 42 | H | 2.0675 | -6.2871 | 0.2003 |
| 43 | H | -0.9663 | 1.2525 | -0.0813 |
| 44 | H | -1.8011 | -0.8991 | 0.5108 |
| 45 | H | -0.855 | -0.3642 | 1.9196 |
| 46 | H | 1.5889 | 0.4307 | 0.8208 |

| | | | | |
|---|---|---|---|---|
| 47 | H | 0.3557 | 1.9736 | -2.2226 |
| 48 | H | 1.359 | 0.7689 | -3.0592 |
| 49 | H | 1.9076 | 1.4361 | -1.5379 |
| 50 | H | -1.6048 | 0.2452 | -2.3887 |
| 51 | H | -0.4729 | -0.8644 | -3.1923 |
| 52 | H | -1.3603 | -1.4059 | -1.7875 |
| 53 | H | -1.1075 | -4.0086 | 5.9241 |
| 54 | H | -0.8327 | -5.7207 | 6.3053 |
| 55 | H | -1.3525 | -5.2479 | 4.6939 |
| 56 | H | 1.5786 | -5.2683 | 7.0871 |
| 57 | H | 2.6755 | -4.3596 | 6.04 |
| 58 | H | 1.3112 | -3.5337 | 6.8025 |

| (8*R*)-**1**,Conf. H | | Standard Orientation (Ångstroms) | | |
|---|---|---|---|---|
| No. | Atom | X | Y | Z |
| 1 | C | -4.6501 | -2.5319 | 3.4274 |
| 2 | C | -5.1588 | -3.2642 | 2.3495 |
| 3 | C | -4.2929 | -3.8128 | 1.4083 |
| 4 | C | -2.9147 | -3.653 | 1.5188 |
| 5 | C | -2.3969 | -2.926 | 2.5939 |
| 6 | C | -3.2627 | -2.3811 | 3.5492 |
| 7 | O | -1.0541 | -2.7045 | 2.8313 |
| 8 | C | -0.1692 | -2.9265 | 1.7962 |
| 9 | C | 0.0057 | -1.9306 | 0.8162 |
| 10 | C | 0.8978 | -2.1781 | -0.2255 |
| 11 | C | 1.6474 | -3.3549 | -0.2511 |
| 12 | C | 1.5205 | -4.3253 | 0.7558 |
| 13 | C | 0.5924 | -4.1178 | 1.7986 |
| 14 | C | -5.5711 | -1.9711 | 4.469 |
| 15 | O | -4.8444 | -4.5161 | 0.3769 |
| 16 | C | 0.4276 | -5.1174 | 2.9307 |
| 17 | C | 1.2533 | -4.7534 | 4.1366 |
| 18 | C | 2.3751 | -5.5613 | 0.6747 |
| 19 | O | 1.1404 | -1.3101 | -1.2552 |
| 20 | C | 0.2137 | -0.2176 | -1.4228 |
| 21 | C | -0.162 | 0.4113 | -0.063 |
| 22 | C | -0.7553 | -0.6321 | 0.8815 |
| 23 | O | 0.985 | 1.0064 | 0.5652 |
| 24 | C | 0.9466 | 0.8126 | -2.2979 |
| 25 | C | -1.0168 | -0.7189 | -2.1978 |
| 26 | C | 0.8318 | -4.4863 | 5.3891 |

| | | | | |
|---|---|---|---|---|
| 27 | C | -0.5994 | -4.5007 | 5.8507 |
| 28 | C | 1.821 | -4.1246 | 6.4669 |
| 29 | H | -6.2299 | -3.4141 | 2.2385 |
| 30 | H | -2.2572 | -4.0969 | 0.7802 |
| 31 | H | -2.8449 | -1.8323 | 4.3908 |
| 32 | H | 2.3497 | -3.5059 | -1.0693 |
| 33 | H | -5.7237 | -2.7039 | 5.2672 |
| 34 | H | -5.1573 | -1.0549 | 4.9034 |
| 35 | H | -6.5426 | -1.7137 | 4.0341 |
| 36 | H | -4.1294 | -4.8504 | -0.1895 |
| 37 | H | 0.729 | -6.1183 | 2.6014 |
| 38 | H | -0.6348 | -5.2297 | 3.1612 |
| 39 | H | 2.3239 | -4.706 | 3.9372 |
| 40 | H | 2.9442 | -5.6981 | 1.5994 |
| 41 | H | 1.7505 | -6.4413 | 0.4919 |
| 42 | H | 3.1015 | -5.4955 | -0.1425 |
| 43 | H | -0.8844 | 1.2238 | -0.2021 |
| 44 | H | -1.798 | -0.824 | 0.6044 |
| 45 | H | -0.7334 | -0.2449 | 1.9072 |
| 46 | H | 1.6728 | 0.3154 | 0.6083 |
| 47 | H | 0.3337 | 1.7036 | -2.4691 |
| 48 | H | 1.208 | 0.3808 | -3.2713 |
| 49 | H | 1.8943 | 1.1217 | -1.8429 |
| 50 | H | -1.7299 | 0.0918 | -2.3798 |
| 51 | H | -0.7184 | -1.1406 | -3.1648 |
| 52 | H | -1.5395 | -1.5231 | -1.6704 |
| 53 | H | -0.8974 | -3.4993 | 6.1787 |
| 54 | H | -0.7137 | -5.1878 | 6.6959 |
| 55 | H | -1.3061 | -4.8188 | 5.0826 |
| 56 | H | 1.7691 | -4.848 | 7.2872 |
| 57 | H | 2.8525 | -4.1128 | 6.0993 |
| 58 | H | 1.5995 | -3.129 | 6.8654 |

| (8*R*)-**1**,Conf. I | | Standard Orientation (Ångstroms) | | |
|---|---|---|---|---|
| No. | Atom | X | Y | Z |
| 1 | C | -4.4266 | -3.7593 | 0.9454 |
| 2 | C | -5.381 | -3.1699 | 1.7825 |
| 3 | C | -4.9761 | -2.4207 | 2.8822 |
| 4 | C | -3.6273 | -2.248 | 3.1714 |
| 5 | C | -2.6655 | -2.8339 | 2.345 |
| 6 | C | -3.0651 | -3.6004 | 1.2433 |

| | | | | |
|---|---|---|---|---|
| 7 | O | -1.3554 | -2.612 | 2.7248 |
| 8 | C | -0.3638 | -2.865 | 1.7987 |
| 9 | C | -0.0822 | -1.9009 | 0.8113 |
| 10 | C | 0.9246 | -2.1754 | -0.1127 |
| 11 | C | 1.6762 | -3.3467 | -0.0137 |
| 12 | C | 1.434 | -4.2875 | 1 |
| 13 | C | 0.3947 | -4.0516 | 1.9254 |
| 14 | C | -4.8552 | -4.6033 | -0.2181 |
| 15 | O | -5.947 | -1.8666 | 3.6639 |
| 16 | C | 0.1134 | -5.019 | 3.0635 |
| 17 | C | 0.926 | -4.7058 | 4.2925 |
| 18 | C | 2.3041 | -5.5133 | 1.0665 |
| 19 | O | 1.2824 | -1.3382 | -1.1347 |
| 20 | C | 0.3748 | -0.2619 | -1.4467 |
| 21 | C | -0.1577 | 0.4089 | -0.1615 |
| 22 | C | -0.8513 | -0.6072 | 0.7434 |
| 23 | O | 0.9069 | 1.0325 | 0.5751 |
| 24 | C | 1.1976 | 0.7451 | -2.2672 |
| 25 | C | -0.7555 | -0.801 | -2.34 |
| 26 | C | 1.7476 | -5.5183 | 4.987 |
| 27 | C | 2.026 | -6.9608 | 4.6677 |
| 28 | C | 2.4809 | -5.0049 | 6.1997 |
| 29 | H | -6.4429 | -3.294 | 1.5851 |
| 30 | H | -3.3069 | -1.6652 | 4.0289 |
| 31 | H | -2.3289 | -4.0842 | 0.6068 |
| 32 | H | 2.4704 | -3.5176 | -0.7384 |
| 33 | H | -4.1277 | -4.54 | -1.0342 |
| 34 | H | -4.9499 | -5.6483 | 0.0925 |
| 35 | H | -5.8177 | -4.2634 | -0.6148 |
| 36 | H | -5.522 | -1.389 | 4.3954 |
| 37 | H | 0.2559 | -6.0432 | 2.7108 |
| 38 | H | -0.9472 | -4.9805 | 3.3377 |
| 39 | H | 0.8038 | -3.6804 | 4.6441 |
| 40 | H | 1.6994 | -6.4194 | 0.9624 |
| 41 | H | 3.0447 | -5.5249 | 0.2593 |
| 42 | H | 2.8543 | -5.5407 | 2.0112 |
| 43 | H | -0.8624 | 1.2108 | -0.4099 |
| 44 | H | -1.854 | -0.8168 | 0.3545 |
| 45 | H | -0.9498 | -0.1853 | 1.7508 |
| 46 | H | 1.5961 | 0.353 | 0.7019 |
| 47 | H | 0.6032 | 1.6236 | -2.5389 |
| 48 | H | 1.5721 | 0.283 | -3.1883 |
| 49 | H | 2.0846 | 1.079 | -1.7172 |

| | | | | |
|---|---|---|---|---|
| 50 | H | -1.4472 | -0.0037 | -2.631 |
| 51 | H | -0.3454 | -1.2528 | -3.2509 |
| 52 | H | -1.3312 | -1.5917 | -1.8484 |
| 53 | H | 1.7656 | -7.5918 | 5.5241 |
| 54 | H | 3.0906 | -7.0979 | 4.4504 |
| 55 | H | 1.4659 | -7.3356 | 3.8091 |
| 56 | H | 3.5624 | -5.0955 | 6.0544 |
| 57 | H | 2.2608 | -3.9521 | 6.4056 |
| 58 | H | 2.1986 | -5.5826 | 7.0861 |

| (8*R*)-**1**,Conf. J | | Standard Orientation (Ångstroms) | | |
|---|---|---|---|---|
| No. | Atom | X | Y | Z |
| 1 | C | -4.5648 | -2.0458 | 3.6659 |
| 2 | C | -4.9735 | -3.0276 | 2.7559 |
| 3 | C | -4.0449 | -3.5912 | 1.8841 |
| 4 | C | -2.7091 | -3.2034 | 1.9044 |
| 5 | C | -2.2881 | -2.2343 | 2.8165 |
| 6 | C | -3.2159 | -1.6655 | 3.6969 |
| 7 | O | -0.9967 | -1.7637 | 2.9532 |
| 8 | C | -0.0409 | -2.214 | 2.0665 |
| 9 | C | 0.0953 | -1.5619 | 0.8252 |
| 10 | C | 1.0536 | -2.0346 | -0.0692 |
| 11 | C | 1.8992 | -3.0841 | 0.2901 |
| 12 | C | 1.8094 | -3.6975 | 1.5503 |
| 13 | C | 0.8266 | -3.2573 | 2.4643 |
| 14 | C | -5.5411 | -1.4523 | 4.6378 |
| 15 | O | -4.4124 | -4.5468 | 0.9816 |
| 16 | C | 0.6951 | -3.881 | 3.8447 |
| 17 | C | -0.0322 | -5.1998 | 3.8278 |
| 18 | C | 2.756 | -4.8241 | 1.8663 |
| 19 | O | 1.2676 | -1.5129 | -1.3163 |
| 20 | C | 0.2471 | -0.6394 | -1.8413 |
| 21 | C | -0.2494 | 0.348 | -0.7624 |
| 22 | C | -0.7843 | -0.3944 | 0.4603 |
| 23 | O | 0.8068 | 1.2282 | -0.3435 |
| 24 | C | 0.9163 | 0.1284 | -2.9934 |
| 25 | C | -0.8916 | -1.4909 | -2.4283 |
| 26 | C | 0.3693 | -6.3875 | 4.3239 |
| 27 | C | 1.6722 | -6.6454 | 5.0278 |
| 28 | C | -0.5139 | -7.6023 | 4.1987 |
| 29 | H | -6.0125 | -3.3436 | 2.7366 |

| | | | | |
|---|---|---|---|---|
| 30 | H | -2.0201 | -3.6749 | 1.211 |
| 31 | H | -2.8767 | -0.918 | 4.4114 |
| 32 | H | 2.643 | -3.4223 | -0.4293 |
| 33 | H | -5.5586 | -2.0434 | 5.5585 |
| 34 | H | -5.2685 | -0.4206 | 4.8838 |
| 35 | H | -6.5501 | -1.4286 | 4.213 |
| 36 | H | -5.3602 | -4.7286 | 1.0893 |
| 37 | H | 0.151 | -3.2068 | 4.5169 |
| 38 | H | 1.6911 | -3.9509 | 4.29 |
| 39 | H | -1.0128 | -5.1533 | 3.3542 |
| 40 | H | 3.2741 | -4.6453 | 2.8131 |
| 41 | H | 2.2153 | -5.7736 | 1.9114 |
| 42 | H | 3.5303 | -4.9229 | 1.0975 |
| 43 | H | -1.0377 | 0.994 | -1.1657 |
| 44 | H | -1.7917 | -0.7686 | 0.2472 |
| 45 | H | -0.8433 | 0.2992 | 1.3077 |
| 46 | H | 1.5578 | 0.6606 | -0.0854 |
| 47 | H | 0.2281 | 0.8461 | -3.4516 |
| 48 | H | 1.2638 | -0.562 | -3.7711 |
| 49 | H | 1.807 | 0.667 | -2.6509 |
| 50 | H | -1.6699 | -0.8621 | -2.8729 |
| 51 | H | -0.5088 | -2.1666 | -3.2021 |
| 52 | H | -1.3592 | -2.1339 | -1.6758 |
| 53 | H | 2.2836 | -5.7509 | 5.1595 |
| 54 | H | 2.2667 | -7.3706 | 4.4623 |
| 55 | H | 1.4848 | -7.0572 | 6.0252 |
| 56 | H | -0.767 | -7.9909 | 5.1906 |
| 57 | H | -1.4525 | -7.3842 | 3.6782 |
| 58 | H | 0.0015 | -8.3885 | 3.6374 |

| (8*R*)-**1**,Conf. K | | Standard Orientation (Ångstroms) | | |
|---|---|---|---|---|
| No. | Atom | X | Y | Z |
| 1 | C | -4.9011 | -1.9313 | 3.2866 |
| 2 | C | -5.4092 | -2.7357 | 2.261 |
| 3 | C | -4.5411 | -3.4339 | 1.4272 |
| 4 | C | -3.1617 | -3.354 | 1.595 |
| 5 | C | -2.6447 | -2.5546 | 2.6183 |
| 6 | C | -3.5136 | -1.8594 | 3.4673 |
| 7 | O | -1.301 | -2.397 | 2.9008 |
| 8 | C | -0.3918 | -2.7634 | 1.9293 |
| 9 | C | -0.1187 | -1.8733 | 0.8724 |

| | | | | |
|---|---|---|---|---|
| 10 | C | 0.7998 | -2.2638 | -0.1002 |
| 11 | C | 1.4789 | -3.4773 | 0.0144 |
| 12 | C | 1.2497 | -4.3448 | 1.0944 |
| 13 | C | 0.2963 | -3.9903 | 2.073 |
| 14 | C | -5.8282 | -1.2077 | 4.2166 |
| 15 | O | -5.0919 | -4.2023 | 0.4429 |
| 16 | C | 0.0375 | -4.8729 | 3.2831 |
| 17 | C | 0.9541 | -4.5477 | 4.4329 |
| 18 | C | 2.0443 | -5.6203 | 1.1739 |
| 19 | O | 1.138 | -1.5076 | -1.1895 |
| 20 | C | 0.288 | -0.3836 | -1.4965 |
| 21 | C | -0.1067 | 0.3851 | -0.2163 |
| 22 | C | -0.8031 | -0.5338 | 0.7853 |
| 23 | O | 1.0465 | 0.9667 | 0.4133 |
| 24 | C | 1.1198 | 0.5205 | -2.4213 |
| 25 | C | -0.9361 | -0.8813 | -2.2838 |
| 26 | C | 1.7729 | -5.3733 | 5.1153 |
| 27 | C | 1.9405 | -6.8455 | 4.8615 |
| 28 | C | 2.6202 | -4.8441 | 6.2441 |
| 29 | H | -6.4819 | -2.826 | 2.1083 |
| 30 | H | -2.5036 | -3.9136 | 0.9405 |
| 31 | H | -3.0991 | -1.255 | 4.2716 |
| 32 | H | 2.2081 | -3.7393 | -0.7506 |
| 33 | H | -6.0656 | -1.8416 | 5.0764 |
| 34 | H | -5.3733 | -0.2786 | 4.576 |
| 35 | H | -6.7596 | -0.9373 | 3.7082 |
| 36 | H | -4.3769 | -4.6407 | -0.0473 |
| 37 | H | 0.0897 | -5.9222 | 2.9832 |
| 38 | H | -0.9962 | -4.7488 | 3.6262 |
| 39 | H | 0.9191 | -3.4994 | 4.7331 |
| 40 | H | 2.7182 | -5.728 | 0.317 |
| 41 | H | 2.6626 | -5.6295 | 2.0759 |
| 42 | H | 1.3782 | -6.4885 | 1.171 |
| 43 | H | -0.7711 | 1.2221 | -0.4602 |
| 44 | H | -1.8419 | -0.6916 | 0.4748 |
| 45 | H | -0.8035 | -0.0568 | 1.7727 |
| 46 | H | 1.6928 | 0.2461 | 0.5382 |
| 47 | H | 0.5705 | 1.4259 | -2.6991 |
| 48 | H | 1.3965 | -0.0112 | -3.3395 |
| 49 | H | 2.0639 | 0.8147 | -1.9493 |
| 50 | H | -1.5896 | -0.0508 | -2.5702 |
| 51 | H | -0.6226 | -1.4048 | -3.1947 |
| 52 | H | -1.5295 | -1.6036 | -1.7142 |

| | | | | |
|---|---|---|---|---|
| 53 | H | 1.7019 | -7.412 | 5.7679 |
| 54 | H | 2.9772 | -7.0624 | 4.5831 |
| 55 | H | 1.3009 | -7.2284 | 4.0643 |
| 56 | H | 3.6813 | -5.0114 | 6.0314 |
| 57 | H | 2.4792 | -3.7699 | 6.4035 |
| 58 | H | 2.3675 | -5.3545 | 7.1792 |

| (8*R*)-**1**,Conf. L | | Standard Orientation (Ångstroms) | | |
|---|---|---|---|---|
| No. | Atom | X | Y | Z |
| 1 | C | -3.1589 | -2.2634 | 3.6315 |
| 2 | C | -4.3733 | -2.9391 | 3.4593 |
| 3 | C | -4.494 | -3.8977 | 2.4571 |
| 4 | C | -3.4218 | -4.2039 | 1.6283 |
| 5 | C | -2.2017 | -3.5474 | 1.7982 |
| 6 | C | -2.072 | -2.5809 | 2.802 |
| 7 | O | -1.2042 | -3.9411 | 0.9278 |
| 8 | C | 0.0039 | -3.2764 | 0.9698 |
| 9 | C | 0.1731 | -2.1266 | 0.1728 |
| 10 | C | 1.4231 | -1.5087 | 0.1606 |
| 11 | C | 2.4665 | -2.0017 | 0.9443 |
| 12 | C | 2.3094 | -3.1557 | 1.7279 |
| 13 | C | 1.066 | -3.8264 | 1.7261 |
| 14 | C | -2.9959 | -1.2576 | 4.7326 |
| 15 | O | -5.6634 | -4.5717 | 2.2581 |
| 16 | C | 0.8569 | -5.1063 | 2.5209 |
| 17 | C | 0.4235 | -4.8485 | 3.9413 |
| 18 | C | 3.497 | -3.6612 | 2.5027 |
| 19 | O | 1.7265 | -0.3978 | -0.5795 |
| 20 | C | 0.8281 | -0.0727 | -1.6599 |
| 21 | C | -0.6435 | -0.1985 | -1.2121 |
| 22 | C | -0.9467 | -1.6013 | -0.6882 |
| 23 | O | -0.9472 | 0.7545 | -0.1791 |
| 24 | C | 1.1458 | 1.3848 | -2.0339 |
| 25 | C | 1.1522 | -0.9608 | -2.8732 |
| 26 | C | -0.5913 | -5.4196 | 4.6211 |
| 27 | C | -1.516 | -6.4777 | 4.0856 |
| 28 | C | -0.8895 | -5.0067 | 6.0391 |
| 29 | H | -5.2119 | -2.7114 | 4.1105 |
| 30 | H | -3.5339 | -4.9575 | 0.8547 |
| 31 | H | -1.1259 | -2.0711 | 2.9612 |
| 32 | H | 3.4248 | -1.4857 | 0.9183 |

| | | | | |
|---|---|---|---|---|
| 33 | H | -2.2922 | -0.4708 | 4.4409 |
| 34 | H | -3.9494 | -0.7708 | 4.9628 |
| 35 | H | -2.6232 | -1.7501 | 5.636 |
| 36 | H | -6.3217 | -4.2423 | 2.8914 |
| 37 | H | 1.7921 | -5.6782 | 2.5446 |
| 38 | H | 0.1605 | -5.7522 | 1.979 |
| 39 | H | 1.0179 | -4.0968 | 4.4595 |
| 40 | H | 3.2403 | -3.8165 | 3.5543 |
| 41 | H | 3.8589 | -4.599 | 2.0697 |
| 42 | H | 4.325 | -2.9442 | 2.4805 |
| 43 | H | -1.3204 | 0.031 | -2.043 |
| 44 | H | -1.0842 | -2.2918 | -1.5286 |
| 45 | H | -1.8796 | -1.566 | -0.1166 |
| 46 | H | -0.2774 | 0.6311 | 0.5196 |
| 47 | H | 0.4935 | 1.7456 | -2.8359 |
| 48 | H | 2.1866 | 1.4836 | -2.3645 |
| 49 | H | 1.0411 | 2.0516 | -1.1708 |
| 50 | H | 0.5169 | -0.7116 | -3.7295 |
| 51 | H | 2.1998 | -0.8383 | -3.1724 |
| 52 | H | 1.0271 | -2.0257 | -2.6532 |
| 53 | H | -2.5516 | -6.1238 | 4.1165 |
| 54 | H | -1.4453 | -7.381 | 4.701 |
| 55 | H | -1.2997 | -6.7704 | 3.0567 |
| 56 | H | -0.8012 | -5.8667 | 6.7109 |
| 57 | H | -0.206 | -4.231 | 6.4001 |
| 58 | H | -1.9078 | -4.6105 | 6.1117 |

| (8*R*)-**1**,Conf. M | | Standard Orientation (Ångstroms) | | |
|---|---|---|---|---|
| No. | Atom | X | Y | Z |
| 1 | C | -3.1185 | -2.178 | 3.7306 |
| 2 | C | -4.338 | -2.8538 | 3.6131 |
| 3 | C | -4.5077 | -3.8158 | 2.6238 |
| 4 | C | -3.4742 | -4.1297 | 1.748 |
| 5 | C | -2.2455 | -3.4743 | 1.8655 |
| 6 | C | -2.0698 | -2.5014 | 2.8564 |
| 7 | O | -1.2872 | -3.8766 | 0.9549 |
| 8 | C | -0.057 | -3.2525 | 0.9769 |
| 9 | C | 0.1341 | -2.1089 | 0.176 |
| 10 | C | 1.4023 | -1.5305 | 0.1405 |
| 11 | C | 2.4436 | -2.056 | 0.9055 |
| 12 | C | 2.2641 | -3.2035 | 1.6937 |

| | | | | |
|---|---|---|---|---|
| 13 | C | 1.0005 | -3.8351 | 1.7149 |
| 14 | C | -2.9108 | -1.1678 | 4.8193 |
| 15 | O | -5.7176 | -4.4417 | 2.5482 |
| 16 | C | 0.7668 | -5.1062 | 2.5172 |
| 17 | C | 0.3658 | -4.8308 | 3.9437 |
| 18 | C | 3.4486 | -3.7443 | 2.4494 |
| 19 | O | 1.7268 | -0.4294 | -0.605 |
| 20 | C | 0.8179 | -0.0723 | -1.6662 |
| 21 | C | -0.648 | -0.1535 | -1.1902 |
| 22 | C | -0.9849 | -1.5472 | -0.6626 |
| 23 | O | -0.9031 | 0.806 | -0.1507 |
| 24 | C | 1.174 | 1.3757 | -2.0424 |
| 25 | C | 1.0899 | -0.9663 | -2.8879 |
| 26 | C | -0.6327 | -5.3949 | 4.6527 |
| 27 | C | -1.5611 | -6.4671 | 4.1542 |
| 28 | C | -0.9064 | -4.9578 | 6.0687 |
| 29 | H | -5.1598 | -2.6348 | 4.2901 |
| 30 | H | -3.6015 | -4.8833 | 0.978 |
| 31 | H | -1.1177 | -1.991 | 2.9718 |
| 32 | H | 3.417 | -1.5701 | 0.8622 |
| 33 | H | -2.4892 | -1.6541 | 5.7045 |
| 34 | H | -2.2296 | -0.3758 | 4.4907 |
| 35 | H | -3.856 | -0.6889 | 5.0956 |
| 36 | H | -5.6946 | -5.0708 | 1.8086 |
| 37 | H | 1.6841 | -5.7066 | 2.5257 |
| 38 | H | 0.0392 | -5.7297 | 1.9901 |
| 39 | H | 0.9666 | -4.068 | 4.438 |
| 40 | H | 3.2035 | -3.8954 | 3.5044 |
| 41 | H | 3.7772 | -4.6907 | 2.0087 |
| 42 | H | 4.2959 | -3.0506 | 2.4166 |
| 43 | H | -1.3331 | 0.0983 | -2.0078 |
| 44 | H | -1.1597 | -2.2314 | -1.5013 |
| 45 | H | -1.9051 | -1.4851 | -0.0726 |
| 46 | H | -0.2237 | 0.6634 | 0.535 |
| 47 | H | 0.5176 | 1.7596 | -2.8302 |
| 48 | H | 2.2106 | 1.4428 | -2.3933 |
| 49 | H | 1.1075 | 2.0424 | -1.1754 |
| 50 | H | 0.446 | -0.6945 | -3.7309 |
| 51 | H | 2.1348 | -0.876 | -3.2074 |
| 52 | H | 0.9355 | -2.0274 | -2.6681 |
| 53 | H | -1.3515 | -6.7881 | 3.1323 |
| 54 | H | -2.5966 | -6.113 | 4.1837 |
| 55 | H | -1.4848 | -7.3535 | 4.7931 |

| | | | | |
|---|---|---|---|---|
| 56 | H | -0.7978 | -5.8043 | 6.7546 |
| 57 | H | -0.2224 | -4.1701 | 6.4015 |
| 58 | H | -1.9262 | -4.5682 | 6.154 |

| (8*R*)-**1**,Conf. N | | Standard Orientation (Ångstroms) | | |
|---|---|---|---|---|
| No. | Atom | X | Y | Z |
| 1 | C | -4.6248 | -2.6794 | 3.3943 |
| 2 | C | -5.1081 | -3.3993 | 2.2953 |
| 3 | C | -4.2175 | -3.8821 | 1.3387 |
| 4 | C | -2.8479 | -3.6696 | 1.4567 |
| 5 | C | -2.356 | -2.9584 | 2.5519 |
| 6 | C | -3.2437 | -2.4766 | 3.5208 |
| 7 | O | -1.0241 | -2.6908 | 2.7996 |
| 8 | C | -0.1184 | -2.9048 | 1.7811 |
| 9 | C | 0.0589 | -1.9093 | 0.8013 |
| 10 | C | 0.9798 | -2.1421 | -0.2187 |
| 11 | C | 1.7528 | -3.3038 | -0.2235 |
| 12 | C | 1.62 | -4.2746 | 0.7821 |
| 13 | C | 0.6644 | -4.0821 | 1.8031 |
| 14 | C | -5.5627 | -2.1847 | 4.4551 |
| 15 | O | -4.6578 | -4.5844 | 0.2545 |
| 16 | C | 0.4911 | -5.0838 | 2.9318 |
| 17 | C | 1.2896 | -4.709 | 4.1533 |
| 18 | C | 2.4952 | -5.497 | 0.7209 |
| 19 | O | 1.2285 | -1.2719 | -1.2455 |
| 20 | C | 0.2739 | -0.2095 | -1.4455 |
| 21 | C | -0.149 | 0.4209 | -0.1004 |
| 22 | C | -0.734 | -0.6293 | 0.8415 |
| 23 | O | 0.9679 | 1.0528 | 0.5467 |
| 24 | C | 0.996 | 0.8333 | -2.3147 |
| 25 | C | -0.9243 | -0.753 | -2.2424 |
| 26 | C | 0.8432 | -4.4579 | 5.4004 |
| 27 | C | -0.5942 | -4.5073 | 5.8391 |
| 28 | C | 1.8073 | -4.0792 | 6.4948 |
| 29 | H | -6.1749 | -3.5782 | 2.1971 |
| 30 | H | -2.1896 | -4.0715 | 0.6935 |
| 31 | H | -2.8468 | -1.9371 | 4.3784 |
| 32 | H | 2.4747 | -3.4444 | -1.0261 |
| 33 | H | -5.6817 | -2.9449 | 5.2332 |
| 34 | H | -5.1838 | -1.2649 | 4.9131 |
| 35 | H | -6.5459 | -1.9548 | 4.0314 |

| | | | | |
|---|---|---|---|---|
| 36 | H | -5.6239 | -4.6674 | 0.3087 |
| 37 | H | 0.812 | -6.0804 | 2.608 |
| 38 | H | -0.5737 | -5.2102 | 3.1426 |
| 39 | H | 2.3618 | -4.6367 | 3.9704 |
| 40 | H | 3.0438 | -5.6264 | 1.6589 |
| 41 | H | 1.889 | -6.3864 | 0.522 |
| 42 | H | 3.2403 | -5.4189 | -0.0782 |
| 43 | H | -0.8897 | 1.2123 | -0.263 |
| 44 | H | -1.7662 | -0.8488 | 0.5463 |
| 45 | H | -0.7413 | -0.2337 | 1.8642 |
| 46 | H | 1.6714 | 0.3791 | 0.6112 |
| 47 | H | 0.3616 | 1.7045 | -2.5082 |
| 48 | H | 1.2912 | 0.4002 | -3.2778 |
| 49 | H | 1.924 | 1.1742 | -1.8422 |
| 50 | H | -1.6549 | 0.0357 | -2.4497 |
| 51 | H | -0.5924 | -1.1768 | -3.1976 |
| 52 | H | -1.4372 | -1.5655 | -1.7179 |
| 53 | H | -0.7051 | -5.197 | 6.6828 |
| 54 | H | -1.2805 | -4.8433 | 5.0602 |
| 55 | H | -0.922 | -3.5136 | 6.162 |
| 56 | H | 1.7598 | -4.808 | 7.3106 |
| 57 | H | 2.8437 | -4.0417 | 6.143 |
| 58 | H | 1.5569 | -3.0912 | 6.8948 |

| (8*R*)-**1**,Conf. O | | Standard Orientation (Ångstroms) | | |
|---|---|---|---|---|
| No. | Atom | X | Y | Z |
| 1 | C | -3.9484 | -3.9039 | 1.5506 |
| 2 | C | -4.9598 | -3.3798 | 2.3652 |
| 3 | C | -4.6537 | -2.3915 | 3.2969 |
| 4 | C | -3.3541 | -1.9207 | 3.4388 |
| 5 | C | -2.3349 | -2.4418 | 2.6409 |
| 6 | C | -2.6332 | -3.4383 | 1.7034 |
| 7 | O | -1.0816 | -1.9099 | 2.8753 |
| 8 | C | -0.0524 | -2.2727 | 2.0316 |
| 9 | C | 0.1018 | -1.5868 | 0.8107 |
| 10 | C | 1.1279 | -1.9817 | -0.0451 |
| 11 | C | 2.0185 | -2.9855 | 0.3357 |
| 12 | C | 1.9094 | -3.6278 | 1.5799 |
| 13 | C | 0.8607 | -3.2663 | 2.4541 |
| 14 | C | -4.2509 | -4.9974 | 0.5685 |
| 15 | O | -5.6147 | -1.855 | 4.1026 |

| | | | | |
|---|---|---|---|---|
| 16 | C | 0.7075 | -3.9186 | 3.8192 |
| 17 | C | 0.0454 | -5.2701 | 3.7565 |
| 18 | C | 2.9111 | -4.6972 | 1.9231 |
| 19 | O | 1.3677 | -1.4226 | -1.2708 |
| 20 | C | 0.3282 | -0.5901 | -1.8243 |
| 21 | C | -0.275 | 0.3399 | -0.7487 |
| 22 | C | -0.8263 | -0.4633 | 0.4277 |
| 23 | O | 0.7078 | 1.2663 | -0.2582 |
| 24 | C | 1.0096 | 0.243 | -2.9225 |
| 25 | C | -0.7319 | -1.4858 | -2.4879 |
| 26 | C | 0.4954 | -6.4505 | 4.2276 |
| 27 | C | 1.8008 | -6.6669 | 4.9411 |
| 28 | C | -0.3304 | -7.7007 | 4.064 |
| 29 | H | -5.9763 | -3.7491 | 2.2658 |
| 30 | H | -3.1347 | -1.1508 | 4.173 |
| 31 | H | -1.8505 | -3.8704 | 1.0848 |
| 32 | H | 2.8155 | -3.2619 | -0.3526 |
| 33 | H | -3.6053 | -4.9187 | -0.3125 |
| 34 | H | -4.0952 | -5.974 | 1.0371 |
| 35 | H | -5.2865 | -4.9343 | 0.2184 |
| 36 | H | -6.4638 | -2.2813 | 3.9018 |
| 37 | H | 0.1066 | -3.281 | 4.4788 |
| 38 | H | 1.6871 | -3.948 | 4.3036 |
| 39 | H | -0.9327 | -5.2555 | 3.2769 |
| 40 | H | 2.428 | -5.6781 | 1.9433 |
| 41 | H | 3.7165 | -4.7434 | 1.1818 |
| 42 | H | 3.3831 | -4.4942 | 2.8888 |
| 43 | H | -1.0782 | 0.9512 | -1.176 |
| 44 | H | -1.798 | -0.8894 | 0.1544 |
| 45 | H | -0.9682 | 0.2039 | 1.2863 |
| 46 | H | 1.4729 | 0.7358 | 0.0344 |
| 47 | H | 0.3063 | 0.9349 | -3.3973 |
| 48 | H | 1.432 | -0.4067 | -3.6983 |
| 49 | H | 1.8517 | 0.819 | -2.5229 |
| 50 | H | -1.5215 | -0.8884 | -2.9553 |
| 51 | H | -0.2752 | -2.1186 | -3.258 |
| 52 | H | -1.1987 | -2.1734 | -1.7755 |
| 53 | H | 1.6205 | -7.1039 | 5.9291 |
| 54 | H | 2.3735 | -5.7506 | 5.0945 |
| 55 | H | 2.4306 | -7.3566 | 4.3695 |
| 56 | H | -0.5758 | -8.1239 | 5.0436 |
| 57 | H | -1.2724 | -7.5126 | 3.538 |
| 58 | H | 0.2259 | -8.4491 | 3.4903 |

| (8*R*)-**1**,Conf. P | | Standard Orientation (Ångstroms) | | |
|---|---|---|---|---|
| No. | Atom | X | Y | Z |
| 1 | C | -4.8878 | -2.0885 | 3.2596 |
| 2 | C | -5.3678 | -2.8833 | 2.212 |
| 3 | C | -4.469 | -3.5201 | 1.359 |
| 4 | C | -3.095 | -3.3882 | 1.5311 |
| 5 | C | -2.6067 | -2.6021 | 2.576 |
| 6 | C | -3.5033 | -1.9665 | 3.4425 |
| 7 | O | -1.2715 | -2.3992 | 2.8666 |
| 8 | C | -0.3409 | -2.7539 | 1.9113 |
| 9 | C | -0.0649 | -1.8606 | 0.8581 |
| 10 | C | 0.883 | -2.2328 | -0.0936 |
| 11 | C | 1.5861 | -3.4306 | 0.0385 |
| 12 | C | 1.3509 | -4.3017 | 1.1142 |
| 13 | C | 0.3686 | -3.9665 | 2.0709 |
| 14 | C | -5.8385 | -1.4265 | 4.2124 |
| 15 | O | -4.9055 | -4.2992 | 0.3269 |
| 16 | C | 0.0986 | -4.856 | 3.2733 |
| 17 | C | 0.98 | -4.5133 | 4.446 |
| 18 | C | 2.166 | -5.5628 | 1.2101 |
| 19 | O | 1.2268 | -1.4712 | -1.1777 |
| 20 | C | 0.3467 | -0.3792 | -1.5132 |
| 21 | C | -0.0942 | 0.3894 | -0.2483 |
| 22 | C | -0.7811 | -0.5398 | 0.7502 |
| 23 | O | 1.0303 | 1.0107 | 0.3962 |
| 24 | C | 1.1661 | 0.5413 | -2.4328 |
| 25 | C | -0.8468 | -0.923 | -2.3169 |
| 26 | C | 1.7981 | -5.3228 | 5.1481 |
| 27 | C | 2.0009 | -6.7912 | 4.8976 |
| 28 | C | 2.6075 | -4.7777 | 6.2967 |
| 29 | H | -6.4387 | -2.9998 | 2.073 |
| 30 | H | -2.4313 | -3.9093 | 0.849 |
| 31 | H | -3.1118 | -1.3695 | 4.2637 |
| 32 | H | 2.3353 | -3.6792 | -0.7112 |
| 33 | H | -6.0546 | -2.0959 | 5.0507 |
| 34 | H | -5.4151 | -0.495 | 4.6025 |
| 35 | H | -6.7784 | -1.1708 | 3.7119 |
| 36 | H | -5.8764 | -4.3147 | 0.3375 |
| 37 | H | 0.1802 | -5.9035 | 2.9737 |
| 38 | H | -0.9455 | -4.7542 | 3.5911 |

| | | | | |
|---|---|---|---|---|
| 39 | H | 0.9168 | -3.466 | 4.7444 |
| 40 | H | 2.7582 | -5.5662 | 2.1295 |
| 41 | H | 1.5162 | -6.4429 | 1.1835 |
| 42 | H | 2.8666 | -5.6533 | 0.3728 |
| 43 | H | -0.7781 | 1.2043 | -0.5123 |
| 44 | H | -1.8107 | -0.7265 | 0.4252 |
| 45 | H | -0.8092 | -0.0558 | 1.7339 |
| 46 | H | 1.6933 | 0.3088 | 0.5395 |
| 47 | H | 0.5936 | 1.4262 | -2.7298 |
| 48 | H | 1.4755 | 0.0095 | -3.3404 |
| 49 | H | 2.092 | 0.87 | -1.9477 |
| 50 | H | -1.52 | -0.116 | -2.6244 |
| 51 | H | -0.5005 | -1.4461 | -3.216 |
| 52 | H | -1.4286 | -1.6569 | -1.7503 |
| 53 | H | 1.3868 | -7.1864 | 4.0866 |
| 54 | H | 1.7539 | -7.3631 | 5.7984 |
| 55 | H | 3.0476 | -6.9869 | 4.6422 |
| 56 | H | 3.6764 | -4.9236 | 6.1094 |
| 57 | H | 2.4414 | -3.7065 | 6.4529 |
| 58 | H | 2.3428 | -5.2935 | 7.2256 |
